# In Situ Ternary Adduct Formation of Yttrium Polyaminocarboxylates Leads to Small Molecule Capture and Activation

**DOI:** 10.1002/chem.202201780

**Published:** 2022-08-22

**Authors:** Ben. J. Tickner, Carlos Platas‐Iglesias, Simon B. Duckett, Goran Angelovski

**Affiliations:** ^1^ Centre for Hyperpolarisation in Magnetic Resonance Department of Chemistry University of York Heslington YO10 5NY United Kingdom; ^2^ MR Neuroimaging agents Max Planck Institute for Biological Cybernetics Tübingen 72076 Germany; ^3^ Centro de Investigacións Científicas Avanzadas (CICA) Departamento de Química Facultade de Ciencias Universidade da Coruña A Coruña 15001 Spain; ^4^ Laboratory of Molecular and Cellular Neuroimaging International Center for Primate Brain Research (ICPBR) Center for Excellence in Brain Science and Intelligence Technology (CEBSIT) Chinese Academy of Sciences (CAS) Shanghai 200031 PR China

**Keywords:** Coordination, hyperpolarisation, SABRE pyruvate, ternary adducts, yttrium

## Abstract

In this work the chemistry of yttrium complexes is exploited for small molecule capture and activation. Nuclear magnetic resonance (NMR) and density functional theory (DFT) studies were used to investigate the in situ formation of solution state ternary yttrium‐acetate, yttrium‐bicarbonate, and yttrium‐pyruvate adducts with a range of polyaminocarboxylate chelates. These studies reveal that [Y(DO3A)(H_2_O)_2_] (H_3_DO3A – 1,4,7,10‐tetraazacyclododecane‐1,4,7‐tricarboxylic acid) and [Y(EDTA)(H_2_O)_*q*_]^−^ (H_4_EDTA – ethylenediaminetetraacetic acid, *q* = 2 and 3) are able to form ternary adducts with bicarbonate and pyruvate. In the latter, unusual decarboxylation of pyruvate to form acetic acid and CO_2_ was observed and further studied using SABRE‐hyperpolarised ^13^C NMR (SABRE – signal amplification by reversible exchange) to provide information about the reaction timescale and lifetime of intermediates involved in this conversion. The work presented demonstrates that yttrium complexes can capture and activate small molecules, which may lead to novel and useful applications of this metal in catalysis and medical imaging.

## Graphical abstract

The in situ formation of ternary adducts of yttrium(III) polyaminocarboxylate complexes with the endogenous molecules pyruvate, bicarbonate or acetate are investigated using ^13^C NMR, SABRE‐hyperpolarised NMR, and DFT. A novel [Y(EDTA)(pyruvate hydrate)(H_2_O)]^2−^ adduct is linked to decomposition of pyruvate to form acetic acid and CO_2_ highlighting the potential of these adducts for capture and activation of small molecules.

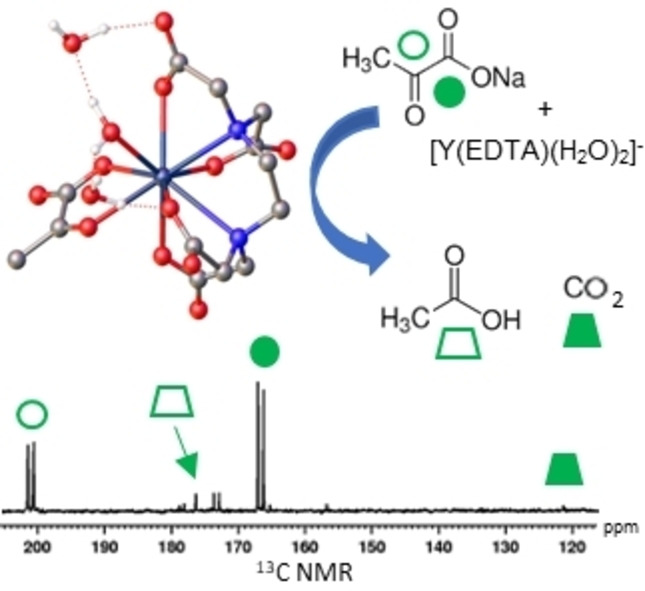

## Introduction

Yttrium is situated in the d‐block of the periodic table and many organoyttrium species have been reported to catalyse a wide range of organic transformations, including hydroaminations, Michael additions, epoxidations and many others.^[1]^ It is also known to form stable complexes in aqueous media with polydentate polyaminocarboxylate ligands in a fashion analogous to many lanthanides.[^2^, ^3^, ^4^, ^5^, ^6^, ^7^, ^8^, ^9^, ^10^] These yttrium coordination compounds have a wide range of medical uses: ^86^Y complexes have been used as positron emission tomography (PET) tracers, while those based on naturally abundant ^89^Y could see applications as hyperpolarised MRI tracers. More widespread is the use of clinically approved radiopharmaceuticals containing the powerful β‐emitter ^90^Y. These usually consist of Y‐DTPA or Y‐DOTA (DTPA: diethylenetriaminepentaacetic acid, DOTA: 1,4,7,10‐tetraazacyclododecane‐1,4,7,10‐tetracetic acid) linked to antibodies or cell‐surface recognition units that target cancerous cells and are used for the treatment of various cancers.^[10]^

These ligands can also coordinate lanthanide ions, in particular Gd(III), which can then be exploited as MRI contrast agents.^[11]^ Their MR relaxation effects are derived predominantly from inner sphere relaxation of ligated water by the paramagnetic Gd(III) centre. Importantly, Y(III) can act as a surrogate for Gd(III) as the two ions have similar ionic radii (90 pm and 93.5 pm respectively). Many studies have therefore used Y(III) analogues to infer information about solution state structure,^[6]^ complexation kinetics,^[12]^ biodistribution, or uptake^[13]^ of Gd(III) complexes. In the context of Gd(III)‐based MRI contrast agents, interactions with various biologically occurring anions can cause a decrease in contrast‐enhancing effectiveness.^[14]^ This arises due to competition of endogenous molecules with bulk water, leading to replacement of the ligated inner‐sphere water molecules, which are then no longer as efficiently relaxed by the Gd(III) centre.[^15^, ^16^] The formation of such adducts is usually confirmed by relaxometry studies in the case of Gd(III) adducts or spectroscopic methods in cases of f‐block elements. In fact, a wide range of lanthanides including Eu(III) and Tb(III) have been reported to form ternary polyaminocarboxylate complexes with amino acids and carbonates.[^17^, ^18^] For example, changes in Eu(III) luminescence upon formation of a ternary adduct with hydrogencarbonate has been used as a route to produce probes sensitive to this endogenous molecule.[^19^, ^20^]

**Figure 1:**
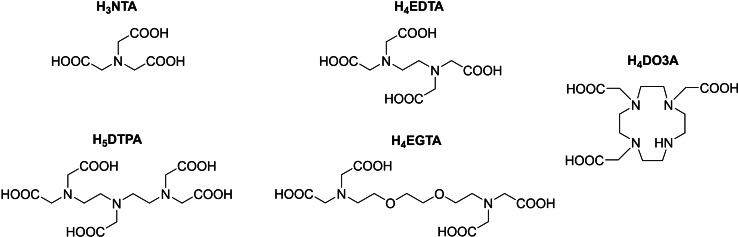
Structures of the polyaminocarboxylate ligands used in this work for formation of yttrium(III) complexes and subsequent investigations of ternary adduct formation with acetate, bicarbonate and pyruvate.

Having these facts in mind, our aim was to establish whether such yttrium(III) species can be used for small molecule capture or activation, as the results would have many implications for their future applications as bioresponsive imaging probes or use in catalysis. To this end, stable yttrium(III) complexes were prepared with the common polyaminocarboyxlate ligands H_3_NTA (nitrilotriacetic acid), H_4_EDTA (ethylenediaminetetraacetic acid), H_5_DTPA, H_4_EGTA (ethylene glycol‐bis(*β*‐aminoethyl ether)‐*N*,*N*,*N*′,*N*′‐tetraacetic acid) and H_3_DO3A (1,4,7,10‐tetraazacyclododecane‐1,4,7‐tricarboxylic acid) (Figure 1). ^13^C NMR spectroscopy was then used to investigate the interaction of these Y(III) complexes with ^13^C‐labelled endogenous molecules acetate, bicarbonate and pyruvate. We chose to focus on these endogenous molecules as they are biologically relevant molecules that can act as markers of disease,[^21^, ^22^] and therefore their capture and interaction with a yttrium (or a lanthanide) probe may be exploited in the future to aid their detection. Furthermore, an exciting route to achieve this could be the use of hyperpolarised ^89^Y MRI probes to discern the presence of such molecules in vivo.^[10]^ Pyruvate is a common metabolite and has been used in vivo as a metabolic imaging probe.^[23]^ Small molecules with similar functionality to pyruvate, such as oxalate, lactate, and citrate have been observed to form ternary adducts with lanthanide complexes containing DO3A‐type ligands[^14^, ^15^, ^24^, ^25^] and examples of solid‐state yttrium‐pyruvate compounds[^26^, ^27^] and analogous compounds with pyruvate derivatives exist.[^28^, ^29^]

To examine the interaction between yttrium complexes and endogenous molecules of interest, ^13^C‐enriched acetate, bicarbonate, or pyruvate were added to the Y(III) complex and indications of in situ ternary adduct formation were assessed by means of ^13^C NMR spectroscopy. DFT was then used to calculate optimised geometries of such adducts in cases where their in situ formation was indicated. Additionally, hyperpolarised ^13^C NMR measurements were used in some cases to boost ^13^C NMR sensitivity and study in situ ternary adduct formation and the reactivity of an yttrium(III) polyaminocarboxylate complex with pyruvate.

## Results and Discussion

### Inertness of [Y(EGTA)(H_2_O)]^−^, [Y(DTPA)(H_2_O)]^2−^ and [Y(NTA)_2_]^3−^ to reaction with acetate, bicarbonate and pyruvate

The formation of yttrium‐acetate adducts in situ was investigated by adding sodium acetate‐[^13^C] to [Y(EGTA)(H_2_O)]^−^, [Y(DTPA)(H_2_O)]^2−^, and [Y(NTA)_2_]^3−^. No additional ^13^C signals were observed, even after reaction at 338 K for 18 h (see Supporting Information, section S1.1).

Similar experiments were performed using sodium bicarbonate‐[^13^C]. Upon reaction with [Y(EGTA)(H_2_O)]^−^ and [Y(NTA)_2_]^3−^, there is a signal between *δ* 160 and 162 corresponding to HCO_3_^−^, which is not broadened and significantly shifted (<1.6 ppm) relative to a reference sample of [^13^C]‐bicarbonate. However, a slight broadening of 14 Hz and a 5.3 ppm downfield shift of this resonance were observed upon reaction between sodium bicarbonate‐[^13^C] and [Y(DTPA)(H_2_O)]^2−^ (see Supporting Information, Figure S3 and Table S2), which may suggest a weak interaction between the two and would be supported by reported examples of Gd(DTPA)‐carbonate ternary complex formation in other systems.^[25]^ When the NMR data involving [Y(EGTA)(H_2_O)]^−^ or [Y(DTPA)(H_2_O)]^2−^ were recorded at lower temperatures, there were no significant changes in the appearance of the NMR spectra (see Supporting Information, Figures S4 and S5, respectively).

Upon reaction of sodium pyruvate‐1‐[^13^C] and [Y(EGTA)(H_2_O)]^−^, [Y(DTPA)(H_2_O)]^2−^, or [Y(NTA)_2_]^3−^ in methanol‐*d*_4_, ^13^C NMR spectroscopy revealed a signal for pyruvate at *δ* 170.5, which was not significantly broadened or shifted relative to that of free sodium pyruvate‐1‐[^13^C] (see Supporting Information, Figure S6). In all cases, there were additional ^13^C NMR signals present at *δ* 176.7 and *δ* 178.4, which were attributed to hemiacetal and hydrate analogues of pyruvate that exist in equilibrium with pyruvate.^[30]^ In the case of [Y(EGTA)(H_2_O)]^−^, additional ^13^C NMR signals appeared at *δ* 179.3 and 182.4, respectively, which are consistent with reported chemical shifts for isomers of parapyruvic acid, a pyruvate dimerisation product that can exist in a variety of keto, enol, open or closed isomers.[^30^, ^31^, ^32^]

### [Y(DO3A)(H_2_O)_2_]: in situ ternary adduct formation with acetate, bicarbonate and pyruvate

**Figure 2:**
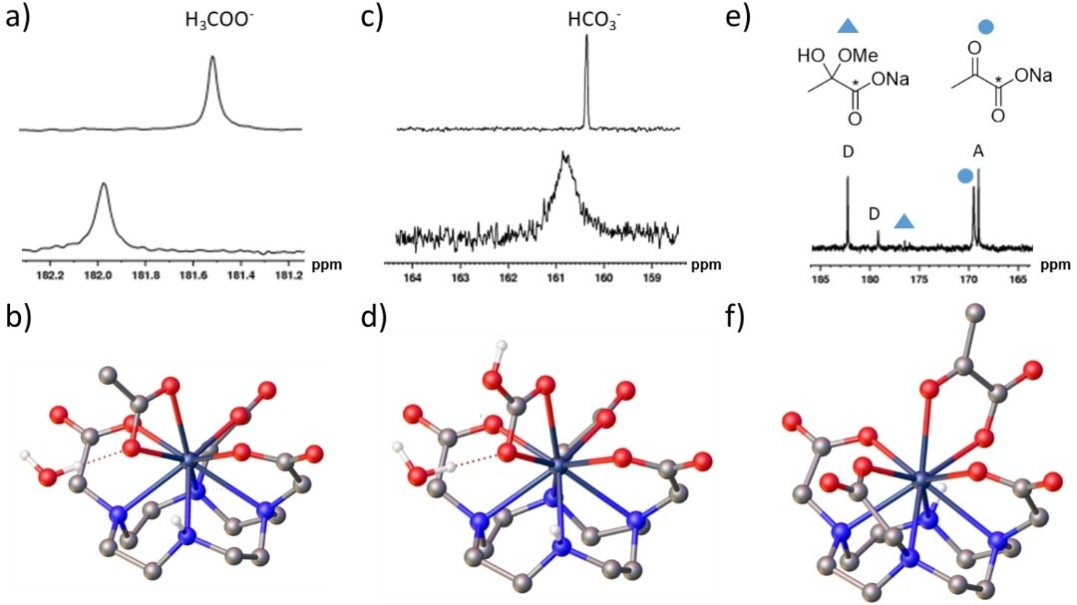
Reactivity of [Y(DO3A)(H_2_O)_2_] with acetate, bicarbonate and pyruvate. Partial ^13^C NMR spectra of a) sodium acetate‐1‐[^13^C] (6 mM) (upper) and [Y(DO3A)(H_2_O)_2_] (4 mM) and sodium acetate‐1‐[^13^C] (1.5 equiv.) (lower) recorded in D_2_O solvent (0.6 mL) at 7 T and 298 K with the same number of scans, processed using the same 3 Hz line broadening parameter and shown on the same vertical scale. b) DFT‐optimised geometry of the [Y(DO3A)(OOCCH_3_)]^−^ adduct formed (see Supporting Information, Figure S9, for more details). Partial ^13^C NMR spectra of c) sodium bicarbonate‐[^13^C] (6 mM) (upper) and [Y(DO3A)(H_2_O)_2_] (4 mM) and sodium bicarbonate‐[^13^C] (1.5 equiv.) (lower) recorded in D_2_O (0.6 mL) at 7 T and 298 K. Spectra are not shown on the same vertical scale. d) DFT‐optimised geometry of [Y(DO3A)(HCO_3_)]^−^ ⋅ H_2_O (see Supporting Information, Figure S11 for more details). e) Partial ^13^C NMR spectra of [Y(DO3A)(H_2_O)_2_] (4 mM) and sodium pyruvate‐1‐[^13^C] (1.5 equiv.) recorded in D_2_O solvent (0.6 mL) at 7 T and 298 K f) DFT‐optimised geometry of [Y(DO3A)(pyruvate)]^‐^ (see Supporting Information, Figure S12 for more details).

The formation of yttrium‐acetate adducts in situ was also investigated by adding sodium acetate‐[^13^C] to [Y(DO3A)(H_2_O)_2_]. This yielded a 0.5 ppm downfield shift and a 3.5 Hz broadening of the ^13^C resonance of sodium acetate‐[^13^C] relative to the expected free ligand resonance (Figure 2a). A smaller 0.02 ppm downfield shift of the ^1^H NMR signal of the acetate CH_3_ group was also observed (see Supporting Information, Figure S8). These results suggest that acetate can form ternary adducts with the yttrium(III) polyaminocarboxylate complexes based on the heptadentate H_3_DO3A, but not the other yttrium complexes based on octadentate ligands such as H_4_EGTA or H_5_DTPA (see above). This is consistent with the known displacement of both coordinated water molecules from [Gd(DO3A)(H_2_O)_2_] by citrate, malonate, bicarbonate, lactate and displacement of only one by phosphate or fluoride.[^14^, ^16^, ^24^, ^33^] We are unaware of previously reported examples of Y‐ or Gd‐containing acetate adducts, although an X‐ray crystal structure of an acetate adduct with a related Yb(III) DO3A‐derived complex has been reported.^[15]^ In this example, acetate displaced both water ligands and we suggest that in our study an analogous [Y(DO3A)(OOCCH_3_)]^‐^ complex is responsible for these observed NMR shifts. DFT calculations performed on [Y(DO3A)(OOCCH_3_))(H_2_O)]^−^ support bidentate binding as displacement of the water molecule to the second coordination sphere leads to a more stable structure, with a relative Gibbs free energy of −12.3 kJ mol^−1^ (Figure 2b and Supporting Information, Figure S9). DFT studies reveal that bidentate coordination is asymmetric, with the axial Y−O bond being considerably longer (2.485 Å) than that involving the oxygen atom in the upper plane of the square antiprismatic coordination environment (2.414 Å), consistent with the labile capping bond effect.^[34]^ Exchange of free acetate and acetate bound in such adducts must be fast on the NMR timescale to observe a single ^13^C NMR signal.

We were also interested to investigate whether yttrium(III) bicarbonate adducts can be formed. Accordingly, sodium bicarbonate‐[^13^C] was added to [Y(DO3A)(H_2_O)_2_] in D_2_O. ^13^C NMR spectroscopy at 298 K and 304 K revealed one broad signal at *δ* 161.46 (HCO_3_^−^) corresponding to HCO_3_^−^, which exhibited a significant 49 Hz line broadening upon reaction with [Y(DO3A)(H_2_O)_2_] (Figure 2c). From these line broadening effects, it is clear that ternary bicarbonate adducts can form between bicarbonate and yttrium‐DO3A complexes. At the pH of these solutions, (7.0–7.5) the equilibrium between HCO_3_^−^ and CO_3_^2−^ favours the former. The fact that no separate ^13^C NMR signals were present for free and ligated HCO_3_^−^ suggests exchange between HCO_3_^−^ and Y⋅⋅⋅HCO_3_^2−^ is fast on the NMR timescale. However, ^13^C NMR spectra collected at lower temperature revealed separate peaks at *δ* 168.6 (CO_3_^2−^) and 161.3 (HCO_3_^−^) in a 1 : 5 intensity ratio at 279 K or 288 K implying exchange between these two forms is now slow (see Supporting Information, Figure S10). DFT calculations support that both inner sphere water molecules are displaced to form [Y(DO3A)(η^2−^HCO_3_)]^‐^ (Figure 2d). Gadolinium(III) carbonate adducts have previously been reported for H_3_DO3A‐derived systems[^6^, ^17^, ^24^, ^35^] and similar bidentate coordination of bicarbonate was observed in the solid state for a Lu(III) complex with the heptadentate polyaminopolycarboxylate ligand H_4_OBETA (2,2′‐oxybis (ethylamine)‐*N*, *N*, *N’*, *N’*‐tetraacetic acid).^[36]^

Evidence for the in situ formation of ternary adducts was more evident upon reaction of [Y(DO3A)(H_2_O)_2_] with sodium pyruvate‐1‐[^13^C]. In addition to ^13^C NMR signals for pyruvate, its adduct, and its dimerisation product(s), a further signal was observed at *δ* 169.2, which does not correspond to reported chemical shifts of parapyruvic acid (*δ* 179–182) or other known pyruvate impurities (Figure 2e).[^30^, ^31^, ^32^] This signal is expected to correspond to a pyruvate molecule ligated to the yttrium(III) centre. As pyruvate contains both keto and carboxylate donor sites, it presents two possible *κ* ^1^ binding modes. *κ* ^2^ ligation involving coordination through both sites is also possible. In fact, examples of pyruvate coordination to metal centres via ketone,^[37]^ carboxylate^[38]^ or *κ* ^2^ modes[^39^, ^40^] have all been previously reported. η^2^ ligation through both oxygen atoms of the carboxylate group could also be possible. Reported X‐ray crystal structures of Yb‐DO3A‐derived lactate and Eu‐DO3A‐derived citrate adducts show *κ* ^2^ coordination with both water molecules displaced.^[15]^ A similar coordination of lactate was evidenced in the case of ytterbium(III) tris‐amide derivatives of H_3_DO3A.^[41]^ DFT calculations were therefore performed to confirm the most likely binding mode and suggest a *κ* ^2^ coordination mode with the replacement of the two coordinated water molecules (Figure 2f). The oxygen atom of the ketone group coordinates at the sterically demanding axial position, with an oxygen atom of the carboxylate group occupying one of the positions of the square antiprismatic coordination polyhedron. The ^13^C chemical shift calculated using DFT for the carboxylate site of pyruvate in [Y(DO3A)(*κ* ^2^‐pyruvate)]^−^ (*δ* 171.0) is in agreement with the experimentally measured value (*δ* 169.2). Therefore, the signal observed at *δ* 169.2 is expected to correspond to [Y(DO3A)(*κ* ^2^‐pyruvate)]^−^. We note that a similar 1.5 ppm upfield ^13^C NMR shift is reported for pyruvate ligated to iridium in an analogous *κ* ^2^ fashion.^[39]^

### Behaviour of [Y(EDTA)(H_2_O)_*q*_]^−^ with bicarbonate and pyruvate: Activation of pyruvate by [Y(EDTA)(H_2_O)_*q*_]^−^

**Figure 3:**
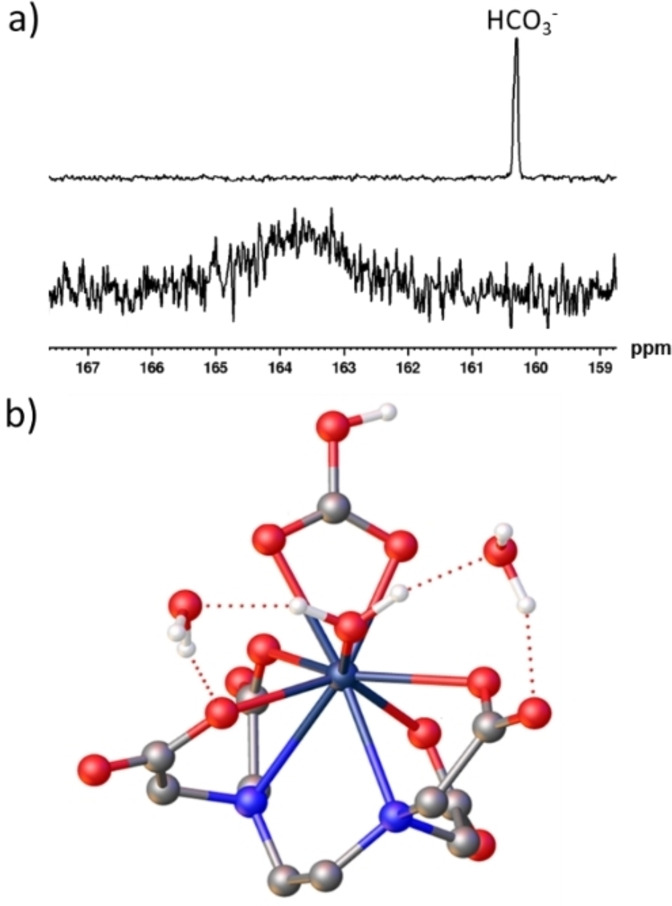
Reactivity of [Y(EDTA)(H_2_O)_*q*_]^−^ with bicarbonate. Partial ^13^C NMR spectra of a) sodium bicarbonate‐[^13^C] (6 mM) (upper) and [Y(EDTA)(H_2_O)_*q*_]^−^ (4 mM) with sodium bicarbonate‐[^13^C] (1.5 equiv.) (lower) recorded in D_2_O (0.6 mL) at 7 T and 298 K. Spectra are not shown on the same vertical scale. c) DFT‐optimised geometries of [Y(EDTA)(HCO_3_)]^2−^ ⋅ 3H_2_O (see Supporting Information, Figure S15 for more details).

No indication of the in situ formation of acetate adducts with [Y(EDTA)(H_2_O)_*q*_]^−^ (*q* = 2 and 3) was observed (see Supporting Information, section S3.1). However, the ability of yttrium(III) complexes containing EDTA to capture and bind bicarbonate was evidenced from ^13^C NMR spectroscopy. This interaction led to a significant 144 Hz line broadening and a 3.4 ppm downfield shift for the bicarbonate ^13^C NMR signal at 298 K (Figure 3a), which broadens further at lower temperature (see Supporting Information, Figure S14). The extent of H^13^CO_3_^−^ NMR line broadening is greater for [(Y(EDTA)(H_2_O)_*q*_]^−^ compared to [Y(DO3A)(H_2_O)_2_] and should be affected by the Y‐bicarbonate exchange rate and affinity constant. The ability of yttrium(III) complexes containing both EDTA^4−^ and DO3A^3−^ to capture and bind bicarbonate is possible and is likely linked to steric effects within the metal inner sphere. Since H_4_EDTA, and H_3_DO3A, are hexa‐ and hepta‐dentate chelators, respectively, they leave sufficient space within the metal coordination sphere for the coordination of at least two additional water molecules.[^4^, ^25^] DFT calculations suggest that an inner sphere water remains coordinated to the metal in [Y(EDTA)(η^2^‐HCO_3_)(H_2_O)]^2−^ (Figure 3b), which is not the case for [Y(DO3A)(η^2−^HCO_3_)]^−^ (Figure 2d).

**Figure 4:**
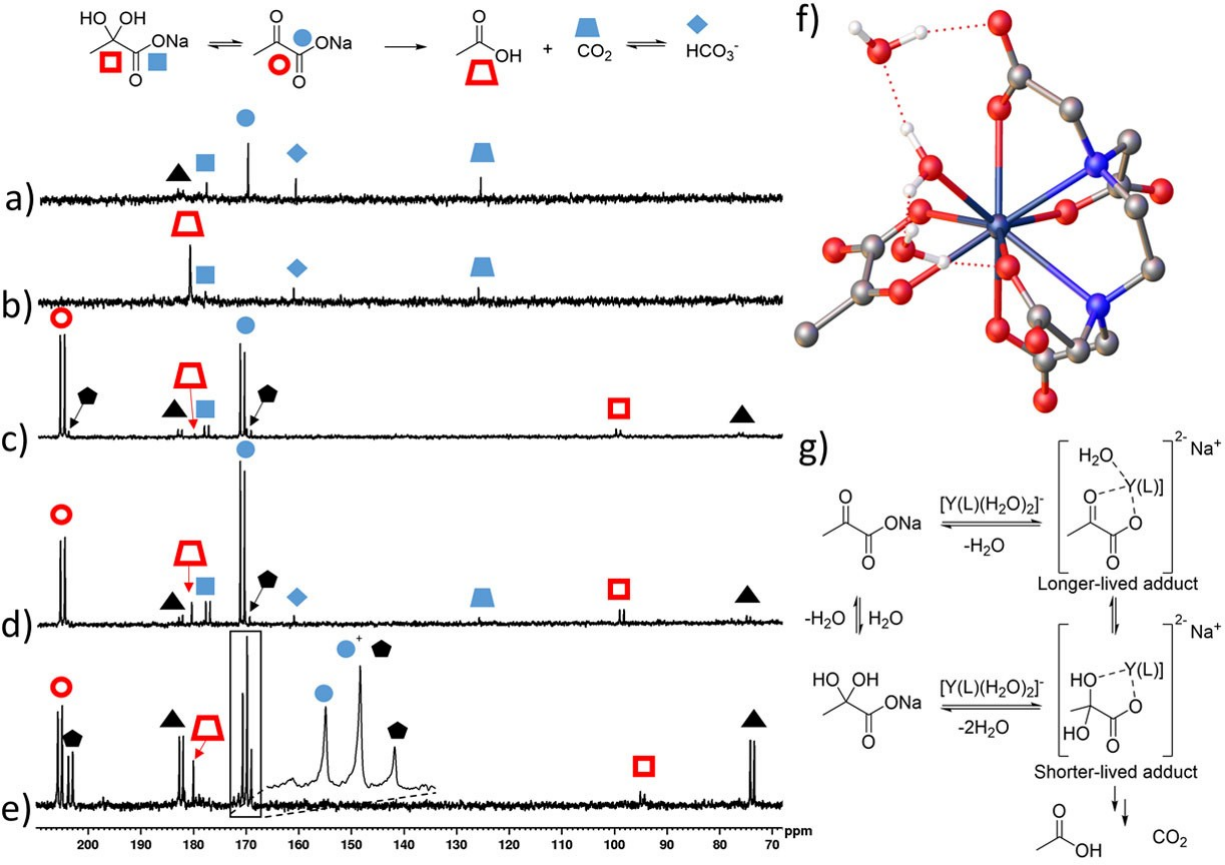
Reactivity of [Y(EDTA)(H_2_O)_*q*_]^−^ with pyruvate and subsequent yttrium(III)‐catalysed pyruvate decomposition. Partial ^13^C NMR spectra of [Y(EDTA)(H_2_O)_*q*_]^−^ (4 mM) and a) sodium pyruvate‐1‐[^13^C] or b) sodium pyruvate‐1,2‐[^13^C_2_] (1.5 equiv.) in methanol‐*d*_4_ after a) 18 h and b) several days at 338 K c)‐d) sodium pyruvate‐1,2‐[^13^C_2_] (1.5 equiv.) in methanol‐*d*_4_ after c) 1 h and d) 3 h at 298 K and e) sodium pyruvate‐1,2‐[^13^C_2_] (1.5 equiv.) in D_2_O after 3 h at 298 K. All spectra were recorded at 298 K and 7 T and are processed with the same 3 Hz line broadening parameter. Spectra were not recorded with the same number of scans and are not shown on the same vertical scale. Signals denoted with squares have been assigned as pyruvate hydrate, although it is possible that these correspond to pyruvate hemiacetal. Signals assigned with the black triangles correspond to an isomer of parapyruvic acid whereas those denoted with a black pentagon are assigned as pyruvate ligated to [Y(EDTA)(*κ* ^2^‐CH_3_C(*O*)CO*O*)(H_2_O)]^2−^ f) DFT‐optimised geometry of [Y(EDTA)(*κ* ^2^‐CH_3_C(*O*)CO*O*)(H_2_O)]^2−^ which contains a coordinated water molecule and two explicit second‐sphere water molecules. g) A summary of yttrium‐catalysed pyruvate decomposition and the proposed adducts involved where L is EDTA.

More interestingly, when sodium pyruvate‐1‐[^13^C] was added to [Y(EDTA)(H_2_O)_*q*_]^−^ in methanol‐*d*_4_ and left for 18 h at 338 K, ^13^C NMR signals could be observed for pyruvate and its hydrate. Unusually, these were accompanied by signals at *δ* 161.4 and 126.3, which correspond to HCO_3_^−^ and CO_2_, respectively (Figure 3a). This experiment was then repeated using sodium pyruvate‐1,2‐[^13^C_2_] instead of sodium pyruvate‐1‐[^13^C], to examine the fate of both carbon sites in this reaction. The analogous ^13^C NMR spectra revealed a signal at *δ* 181.1, corresponding to acetic acid in addition to those of HCO_3_^−^ and CO_2_ (Figure 4b). The presence of these signals, which are not observed in separate solutions of the sodium pyruvate or [Y(EDTA)(H_2_O)_*q*_]^−^ starting materials, suggests that yttrium‐mediated pyruvate activation has occurred. It is expected that in situ formation of ternary [Y(EDTA)(H_2_O)_*q*_]^−^ pyruvate adduct(s) precedes cleavage of the pyruvate C−C bond into CO_2_ and acetic acid. Equilibration of CO_2_ and H_2_O then forms HCO_3_^−^ and H^+^. Similar oxidative cleavage of pyruvate to form acetate and CO_2_ has been reported using lead complexes and water as an oxidising agent: it is expected that a related Criegee‐type mechanism is occurring here.^[42]^ Such reactions typically require oxidising agents such as H_2_O_2_ ^[43]^ or enzyme catalysis;^[44]^ indeed the mild conditions and low‐cost, readily available, catalyst used here may expand the palette of systems that can perform these types of transformations.

When ^13^C NMR spectra were collected after just a few hours of reaction between [Y(EDTA)(H_2_O)_*q*_]^−^ and sodium pyruvate‐1,2‐[^13^C_2_] at 298 K, signals at *δ* 170.1 and 204.7 (^1^ *J*_CC_=62 Hz) were observed, the latter signal overlapping with the free pyruvate signal (Figure 4c–d). This species could not be observed when analogous ^13^C NMR spectra of the starting materials were recorded and were not visible after the reaction has progressed for several days (Figure 4a‐b). Similar ^13^C NMR signals were present at short reaction times when this transformation was performed in D_2_O (Figure 4e). These ^13^C NMR signals are expected to correspond to pyruvate coordinated within the [Y(EDTA)(*κ* ^2^‐CH_3_C(*O*)CO*O*)(H_2_O)]^2−^ adduct and DFT calculations show reasonable agreement between these experimental values (*δ* 170.1 and 204.7) and calculated chemical shifts of *δ* 171.4 and 214.0 for this adduct. Nevertheless, this species was challenging to characterise using 2D NMR studies, X‐ray crystallography, or mass spectrometry. The corresponding chemical shifts calculated for an analogous adduct with pyruvate hydrate were *δ* 107.3 and 181.6, which were not observed in NMR spectra (Figure 4). This suggests that both pyruvate and its hydrate can coordinate to the yttrium(III) EDTA complex and therefore both forms may play a role in pyruvate decomposition.

It is worth noting that this transformation does not occur when sodium pyruvate and YCl_3_ ⋅ xH_2_O or Y(NO_3_)_3_ ⋅ xH_2_O salts were employed (see Supporting Information, section S3.4), indicating that small amounts of free unchelated yttrium(III) ions in solution play no role in this process. In fact, reaction of these salts yields formation of white precipitates, which are expected to be yttrium(III) pyruvate salts (such as [Y(pyruvate)_3_ ⋅ xH_2_O][^26^, ^27^]); consequently, no formation of acetic acid, CO_2_ or HCO_3_^−^ products could be detected. It is also important to note that no clear evidence for the formation of yttrium‐bicarbonate adducts (i. e. no significant broadening of the bicarbonate ^13^C resonance) was observed during these reactions. This could be linked to pyruvate outcompeting bicarbonate for yttrium binding sites, although adduct formation between yttrium and the bicarbonate reaction products at some reaction timepoints cannot be ruled out.

### Probing pyruvate activation by [Y(EDTA)(H_2_O)_*q*_]^−^ using SABRE‐hyperpolarised ^13^C NMR

The ^13^C NMR measurements used to study pyruvate reaction with [Y(EDTA)(H_2_O)_*q*_]^−^ can take many hours for sufficient signal averaging to generate observable resonances. Therefore, it is challenging to determine the timescale over which pyruvate decarboxylation occurs from these experiments. Hyperpolarisation offers a method to improve the sensitivity of NMR by creating molecules in a non‐Boltzmann spin state.^[45]^ As a consequence, NMR signals enhanced by many orders of magnitude can be generated. This is of great use for making low concentration molecules, such as reaction intermediates, visible to NMR.[^43^, ^46^] One such hyperpolarisation technique is SABRE (signal amplification by reversible exchange), which uses iridium complexes to catalytically transfer latent magnetism from parahydrogen (*p*H_2_) to a molecule of interest.[^47^, ^48^] SABRE has been used to produce hyperpolarised pyruvate with ^13^C NMR signals enhanced by up to three orders of magnitude at 9.4 T[^39^, ^49^] and these enhanced signals have been used to monitor a related pyruvate decarboxylation upon reaction with hydrogen peroxide.^[43]^ These enhanced NMR signals have a finite lifetime: they can no longer be observed after hyperpolarised molecules relax back to their thermally populated state. Here, SABRE hyperpolarisation provides a clear advantage, as it is reversible, thus allowing hyperpolarised molecules to be easily regenerated by simply repeating the shaking process with fresh *p*H_2_. Consequently, SABRE‐hyperpolarised NMR was used to study the reaction of pyruvate with [Y(EDTA)(H_2_O)_*q*_]^−^ by preparing ^13^C‐pyruvate in an enhanced spin state before adding [Y(EDTA)(H_2_O)_*q*_]^−^ and observing hyperpolarised signals for any reaction products or short‐lived intermediates.

**Figure 5:**
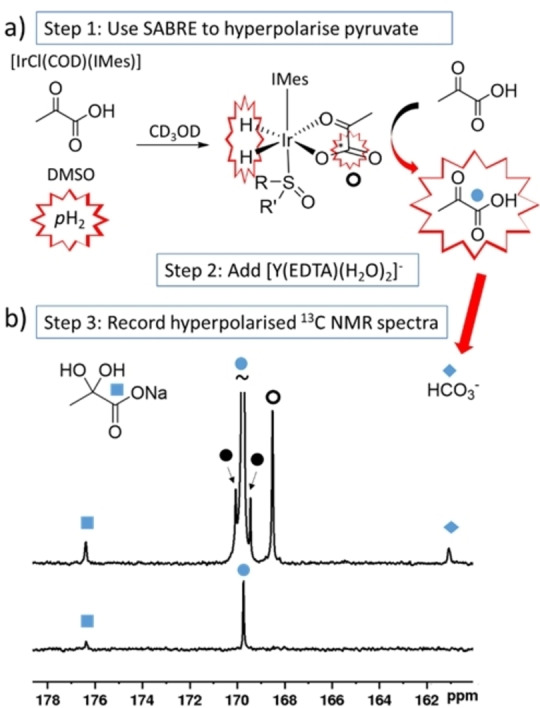
Using SABRE to study interaction between pyruvate and [Y(EDTA)(H_2_O)_*q*_]^−^ a) Depiction of the SABRE process. A solution of [IrCl(COD)(IMes)], DMSO, sodium pyruvate‐1‐[^13^C], and H_2_ are prepared in methanol‐*d*_4_ (0.5 mL) to synthesise the [Ir(H)_2_(*κ* ^2^‐pyruvate)(DMSO)(IMes)] catalyst required to transfer magnetisation from *p*H_2_ to pyruvate ^13^C sites in situ. This is achieved when the NMR tube is shaken with *p*H_2_ (3 bar) for 30 seconds in a mu metal shield. At this point [Y(EDTA)(H_2_O)_*q*_]^−^ is added to the solution containing hyperpolarised pyruvate and single scan ^13^C NMR spectra can be recorded. b) Partial single scan ^13^C NMR spectra at 9.4 T and 298 K immediately after [Y(EDTA)(H_2_O)_*q*_]^−^ (3 mM) in methanol‐*d*_4_ (0.1 mL) is added to a solution of SABRE hyperpolarised sodium pyruvate‐1‐[^13^C] (final concentrations: 30 mM) with [IrCl(COD)(IMes)] (5 mM), dimethyl sulfoxide (30 mM) and [Y(EDTA)(H_2_O)_*q*_]^−^ (3 mM) in methanol‐*d*_4_ (0.5 mL) shaken with *p*H_2_ (3 bar) for 30 seconds in a mu metal shield (above). A single scan thermally polarised spectrum of the same sample taken a few minutes later is shown for comparison (below). Signals marked by the solid black circles correspond to naturally abundant sodium pyruvate‐1,2‐[^13^C_2_].

To achieve this, the SABRE hyperpolarisation method was first used to produce pyruvate with enhanced ^13^C NMR signals by shaking a solution of [IrCl(COD)(IMes)] (COD: *cis*,*cis*‐1,5‐cyclooctadiene, IMes: 1,3‐bis(2,4,6‐trimethyl‐phenyl)imidazol‐2‐ylidene), DMSO, and sodium pyruvate‐1‐[^13^C] in methanol‐*d*_4_ with *p*H_2_ for 30 seconds in a mu metal shield (Figure 5a).[^39^, ^49^] This is necessary to form the [Ir(H)_2_(*κ* ^2^‐pyruvate)(DMSO)(IMes)] catalyst required to transfer latent magnetism from *p*H_2_ to pyruvate and more details of this process have been published elsewhere.[^39^, ^49^] Furthermore, the microtesla magnetic fields provided by the mu metal shield allow spontaneous hyperpolarisation of ^13^C sites.^[50]^ Immediately after *p*H_2_ shaking, the pressure valve sealing the NMR tube was removed and [Y(EDTA)(H_2_O)_*q*_]^−^ dissolved in methanol‐*d*_4_ was added. The tube was then shaken for 1–2 seconds to allow for sufficient reagent mixing before being removed from the mu metal shield and quickly transferred into a 9.4 T spectrometer for analysis by single scan ^13^C NMR.

The obtained SABRE‐hyperpolarised ^13^C NMR spectra revealed enhanced signals for pyruvate‐1‐[^13^C] and the activation product HCO_3_^−^ (Figure 5b). These results, which were collected in just a few seconds, confirm that pyruvate capture and activation occurred almost immediately upon addition of pyruvate to [Y(EDTA)(H_2_O)_*q*_]^−^ and did not require a significant (i. e. many hours) reaction time. When analogous single scan thermally polarised spectra were collected, ^13^C NMR signals for these products could not be observed (Figure 5b), reflecting the magnitude of the NMR sensitivity gain provided by SABRE. Enhanced HCO_3_^−^ signals were not observed when this process was repeated without addition of [Y(EDTA)(H_2_O)_*q*_]^−^ (see Supporting Information, Figure S20a), which confirmed that the iridium catalyst required for SABRE is not responsible for this transformation.[^39^, ^49^] Control measurements were performed to show that HCO_3_^−^ product was not hyperpolarised in a SABRE‐type process via reversible binding to the iridium SABRE catalyst under these conditions (see Supporting Information, Section S4.1).^[51]^ This showed that the enhanced bicarbonate NMR signals were due to its rapid formation from hyperpolarised pyruvate during the lifetime of the hyperpolarised signals (on the order of tens of seconds).

Interestingly, ^13^C NMR signals for the [Y(EDTA)(*κ* ^2^‐pyruvate)(H_2_O)]^2−^ adduct were not observed in these hyperpolarised measurements. In fact, no ^13^C NMR signals for any intermediates were observed, suggesting that those responsible for this decomposition must have a short lifetime. As [Y(EDTA)(*κ* ^2^‐pyruvate)(H_2_O)]^2−^ could be observed using thermally polarised ^13^C NMR spectroscopy (Figure 5), it should exist for sufficient time for it to be observed in experiments that take > tens of minutes to acquire. The fact that the decomposition of pyruvate occurs over shorter timescales (seconds) suggests that intermediates involved in this process are much shorter‐lived. Collectively, these observations suggest that [Y(EDTA)(*κ* ^2^‐pyruvate)(H_2_O)]^2−^ is a resting state with some other short‐lived adduct responsible for pyruvate decomposition, which could be a hydrate adduct (see Supporting Information, Figure S18), not observed in thermally polarised NMR. This would be consistent with a Criegee‐type oxidative cleavage, which has been observed for diol reagents, or pseudo‐diols (such as pyruvate hydrate) that are formed as addition products of *α*‐keto acids.^[42]^

Finally, when the *p*H_2_ shaking step was repeated using fresh *p*H_2_, the enhanced ^13^C NMR signals for pyruvate and HCO_3_^−^ were again observed. These signals were visible when *p*H_2_ shaking was performed in the first 90 mins of reaction, while after this time point, they were no longer observed due to decomposition of the [Ir(H)_2_(κ^2^‐pyruvate)(DMSO)(IMes)] catalyst necessary to produce the SABRE effect.^[49]^ These results illustrate that while pyruvate activation occurs rapidly (i. e. seconds‐minutes), unreacted pyruvate continues to be activated during at least the first 90 minutes of reaction time. This is consistent with a rapid rate of reaction between pyruvate hydrate and [Y(EDTA)(H_2_O)_*q*_]^−^, but a much slower equilibration rate between pyruvate hydrate and pyruvate,^[52]^ which results in rapid activation occurring over a long timescale. The presence of additional byproducts of this process (such as H_2_) could not be confirmed; hence, further mechanistic studies to rationalise this transformation will be highly beneficial.

## Conclusions

In this work we demonstrated that ternary yttrium(III) polyaminocarboxylate acetate, bicarbonate, and pyruvate adducts can be formed using the heptadentate H_3_DO3A ligand, while bicarbonate and pyruvate adducts can also be formed using [Y(EDTA)(H_2_O)_*q*_]^−^ (Table 1). These effects are consistent with reported Gd(III) analogues and correlate with the steric effects of the ligands used. Such ternary adducts exist in equilibrium with the parent yttrium polyaminocarboxylate complex and free endogenous molecule. This is evidenced by ^13^C NMR data for yttrium acetate and yttrium bicarbonate adducts, which exhibit a single exchange broadened peak indicative of rapid exchange between the two forms, hampering isolation and further characterisation of ternary adducts. Notably, the reaction between [Y(EDTA)(H_2_O)_*q*_]^−^ and sodium pyruvate yielded acetic acid, CO_2_ and HCO_3_^−^ as products, suggesting that ternary yttrium(III) adducts are able to activate pyruvate. The distinct behavior of [Y(EDTA)(H_2_O)_*q*_]^−^ in catalysing pyruvate decomposition is likely related to the more open structure of this complex compared to [Y(DO3A)(H_2_O)_2_], which allows simultaneous coordination of pyruvate and a water molecule. SABRE‐hyperpolarised ^13^C NMR has shown that this reaction occurs over short timescales and must involve an intermediate at low concentration with a short lifetime. The reactivity features of Y(III) complexes revealed in this work may have applications in small molecule activation and wider catalysis in the future, as a greater variety of yttrium‐containing systems may be shown to undergo similar reactivity. Moreover, such behavior may be extended to other structurally related substrates. Finally, formation of these yttrium adducts with biologically relevant endogenous molecules may also yield interesting implications for the development of hyperpolarised ^89^Y NMR probes, leading to novel methods for sensing such molecules in vitro and in vivo in the future and hence useful applications in contemporary molecular imaging.

**Table 1:** A summary of ternary adduct formation between the indicated yttrium complex and endogenous molecule. **‘Yes**’ indicates that evidence for adduct formation was observed whereas ‘No’ indicates that clear evidence for adduct formation was not discerned.

Complex	Acetate	Bicarbonate	Pyruvate
[Y(EGTA)(H_2_O)] [Y(DTPA)(H_2_O)]^2−^ [Y(NTA)_2_]^3^ [Y(DO3A)(H_2_O)_2_] [Y(EDTA)(H_2_O)_*q*_]^−^	No No No **Yes** No	No **Yes** No **Yes Yes**	No No No **Yes Yes**

## Experimental Section

**General Remarks**: Commercially available reagents and solvents were used with no further purification. All thermally polarised NMR data were acquired on a Bruker Avance III 300 MHz spectrometer using deuterium lock frequency and processed with TopSpin 3.6 (Bruker GmbH). Hyperpolarised NMR spectra were acquired on a Bruker Avance III 400 MHz spectrometer. Supporting Information‐LRMS was performed on an ion trap SL1100 system (Agilent, Germany).

**General procedure for the synthesis of yttrium polyaminocarboxylate complexes**: The ligands H_3_NTA, H_4_EDTA, H_5_DTPA and H_4_EGTA were all purchased commercially. H_3_DO3A was synthesised according to literature procedures.^[53]^ Syntheses of all the starting yttrium polyaminocarboxylate complexes have been reported previously (see Ref no. [5] for a typical example). Briefly, these ligands (1–3 mmol) were suspended in H_2_O (12 mL). In the case of H_3_NTA, 7 mmol was used. A solution of YCl_3_ ⋅ xH_2_O (1 equiv., assuming x=4) was dissolved in H_2_O (5 mL) and added dropwise, while the pH was maintained at 7.0–7.5 using 0.1 M NaOH or HCl. The mixture was stirred at 60 °C for 18 h. Any excess metal was removed by addition of Chelex 100 to the mixture and stirring at 40 °C for a further 18 h. The reaction mixture was cooled and filtered before Xylenol Orange indicator was used to confirm that no free metal was present. Solvent was then removed by rotary evaporation to yield the desired complex. The complexes were found spectroscopically (^1^H, ^13^C NMR and LRMS) identical to previously reported compounds (see Ref. [7] for [Y(EGTA)(H_2_O)]^−^, Ref. [10] for [Y(DTPA)(H_2_O)]^2−^, Ref. [9] for [Y(NTA)_2_]^3−^, Refs. [8] and [9] for [Y(EDTA)(H_2_O)_*q*_]^−^, and Ref. no [5] for [Y(DO3A)(H_2_O)_2_], MS data for all yttrium complexes is given in Ref. no [4]).

**General procedure for in situ formation of ternary yttrium polyaminocarboxylate adducts**: The yttrium polyminocarboxylate complexes (4 mM) and either sodium pyruvate‐1‐[^13^C], sodium pyruvate‐1,2‐[^13^C_2_], sodium bicarbonate‐[^13^C], or sodium acetate‐1‐[^13^C] (1.5 equiv.) were dissolved in D_2_O or methanol‐*d*_4_ (0.6 mL) and stirred for 18 h at 40 °C.

**DFT calculations**: The structures of the yttrium complexes were optimized with density functional theory (DFT) calculations using the M11 exchange‐correlation functional.^[54]^ In these calculations the quasi‐relativistic ECP28MWB effective core potential and its associated (8s7p6d2f1g)/[6s5p3d2f1g] basis set for yttrium were employed.^[55]^ All other atoms (HCN and O) were described by the standard 6‐311G(d,p) basis set. Frequency calculations were used to confirm that the optimised structures corresponded to local energy minima on the associated potential energy surfaces. All calculations using the ECP approximation were carried out with the Gaussian16 software package.^[56]^ The integration grid was augmented from the default values with the integral=ultrafine keyword in G16. Solvent effects were incorporated using a polarized continuum model using the default stings implemented in G16 [scrf=(pcm,solvent=water].^[57]^ The ^13^C NMR shielding tensors were calculated with the ORCA program package (Version 4.2.1),[^58^, ^59^] using the GIAO method,[^60^, ^61^] and the TPSSh functional.^[62]^ Relativistic effects were considered with the DKH2 method.[^63^, ^64^] These calculations used the old‐DKH‐TZVP basis set, which was obtained by re‐contraction of the TZVPPAll basis set of Ahlrichs.^[65]^ The resolution of identity and chain of spheres exchange (RIJCOSX) approximation was used to speed up the calculation of the NMR chemical shielding constants,[^66^, ^67^] using the Autoaux procedure to generate auxiliary basis sets.^[68]^ The size of the COSX grid used for numerical chain‐of‐sphere integration was increased with the GridX6 and NoFinalGridX keywords. Bulk solvent effects (water) were considered with a continuum model of the solvent defined by the bulk dielectric constant and atomic surface tensions (SMD).^[69]^

**Hyperpolarised measurements**: *Para*‐hydrogen (*p*H_2_) was produced by passing hydrogen gas over a spin‐exchange catalyst (Fe_2_O_3_) at 28 K and used in the hyperpolarisation experiments. This method produces *p*H_2_ with ca. 99 % purity, which is retained at room temperature. The shake & drop method was employed for recording hyperpolarised NMR spectra. Samples were prepared containing [IrCl(COD)(IMes)] (final concentration 5 mM), DMSO (30 mM), sodium pyruvate‐1‐[^13^C_2_] (30 mM) and [Y(EDTA)(H_2_O)_*q*_]^−^ (3 mM) in methanol‐*d*_4_ (0.5 mL) in a 5 mm NMR tube that was fitted with a J. Young's tap. Solutions were subsequently degassed by two freeze‐pump‐thaw cycles using a Schlenk line before reacting with H_2_ (3 bar) for 30 mins at room temperature to form [Ir(H)_2_(*κ* ^2^‐pyruvate)(DMSO)(IMes)] in situ, which was indicated by a colour change from pale yellow to colourless and was confirmed from characteristic ^1^H NMR hydride resonances.[^39^, ^49^] The H_2_ atmosphere was then replaced with *p*H_2_ (3 bar) and shaken vigorously for 30 seconds in a mu metal shield. The lid was quickly removed from the NMR tube whilst it was in the mu metal shield, and a solution of [Y(EDTA)(H_2_O)_*q*_]^−^ (final concentration of 3 mM) in methanol‐*d*_4_ (0.1 mL) added before being recapped and placed quickly into the spectrometer for analysis by ^13^C NMR spectroscopy.

## Conflict of interest

The authors declare no conflict of interest.

1

## Supporting information

As a service to our authors and readers, this journal provides supporting information supplied by the authors. Such materials are peer reviewed and may be re‐organized for online delivery, but are not copy‐edited or typeset. Technical support issues arising from supporting information (other than missing files) should be addressed to the authors.

Supporting Information

## Data Availability

The data that support the findings of this study are available in the supplementary material of this article. Raw NMR data associated with this manuscript can be retrieved and downloaded from the University of York data repository using the following link: https://doi.org/10.15124/08fe7fb7‐fc05‐4c3a‐865a‐95c4be62782c.
