# Supplementary material for: In Situ Ternary Adduct Formation of Yttrium Polyaminocarboxylates Leads to Small Molecule Capture and Activation
Source: Chemistry. 2022 Aug 22;28(57):e202201780. doi: 10.1002/chem.202201780 (PMC9804984; doi:10.1002/chem.202201780)
Supplement: Supplementary file 1 — Supporting Information [file CHEM-28-0-s001.pdf]

Chemistry–A European Journal

Supporting Information

In Situ Ternary Adduct Formation of Yttrium Polyaminocarboxylates Leads to Small Molecule Capture and Activation

Ben. J. Tickner, Carlos Platas-Iglesias, Simon B. Duckett, and Goran Angelovski*

Electronic Supporting Information

In Situ Ternary Adduct Formation of Yttrium Polyaminocarboxylates Leads to Small Molecule Capture and Activation

Ben. J. Tickner,[1,2] Carlos Platas-Iglesias,[3] Simon B. Duckett,[1] and Goran Angelovski*[2,4]

[1] Centre for Hyperpolarisation in Magnetic Resonance, Department of Chemistry, University of York, YO10 5NY, United Kingdom

[2] MR Neuroimaging Agents, Max Planck Institute for Biological Cybernetics, Tuebingen, 72076, Germany

[3] Centro de Investigacións Científicas Avanzadas (CICA), and Departamento de Química, Facultade de Ciencias, Universidade da Coruña, A Coruña, 15001, Spain

[4] Laboratory of Molecular and Cellular Neuroimaging, International Center for Primate Brain Research (ICPBR), Center for Excellence in Brain Science and Intelligence Technology (CEBSIT), Chinese Academy of Sciences (CAS), Shanghai 200031, PR China

*Correspondence to: goran.angelovski@icpbr.ac.cn

Table of Contents

S1. Inertness of [Y(EGTA)(H_2O)]-, [Y(DTPA)(H_2O)]$^{2-}$ and [Y(NTA)$_2$]$^{3-}$ to reaction with acetate, bicarbonate and pyruvate S2

S1.1. Reaction with acetate S2

S1.2. Reaction with bicarbonate S4

S1.3. Reaction with pyruvate S7

S2. [Y(DO3A)(H_2O)$_2$]: in situ ternary adduct formation with acetate, bicarbonate and pyruvate S9

S2.1. Reaction with acetate S9

S2.2. Reaction with bicarbonate S10

S2.3. Reaction with pyruvate S12

S3. Behaviour of [Y(EDTA)(H_2O)$_q$]$^-$ with bicarbonate and pyruvate: Activation of pyruvate by [Y(EDTA)(H_2O)$_q$]$^-$ S13

S3.1. Reaction with acetate S13

S3.2. Reaction with bicarbonate S14

S3.3. Reaction with pyruvate S16

S3.4. Control experiments: Reaction of yttrium salts with pyruvate S18

S4. Probing pyruvate activation by [Y(EDTA)(H_2O)$_q$]$^-$ using SABRE-hyperpolarised ^{13}C NMR S19

S4.1. Control Experiments: SABRE hyperpolarisation of disodium bicarbonate-[^{13}C] S21

S5. References S22

S1. Inertness of $[Y(EGTA)(H_2O)]^-$, $[Y(DTPA)(H_2O)]^{2-}$ and $[Y(NTA)_2]^{3-}$ to reaction with acetate, bicarbonate and pyruvate

S1.1. Reaction with acetate

The complexes $[Y(EGTA)(H_2O)]^-$, $[Y(DTPA)(H_2O)]^{2-}$ or $[Y(NTA)_2]^{3-}$ (4 mM) and sodium acetate-1-[^{13}C] (1.5 equiv.) were dissolved in D_2O (0.6 mL) and left at 40 °C for 18 hours. ^{13}C, and to a lesser extent ^1H, NMR spectroscopy was used to examine the appearance of additional NMR signals, or signal broadening, to provide evidence for the *in situ* formation of ternary adducts. These spectra are shown in Figure S1 and S2 respectively and are summarised in Table S1.

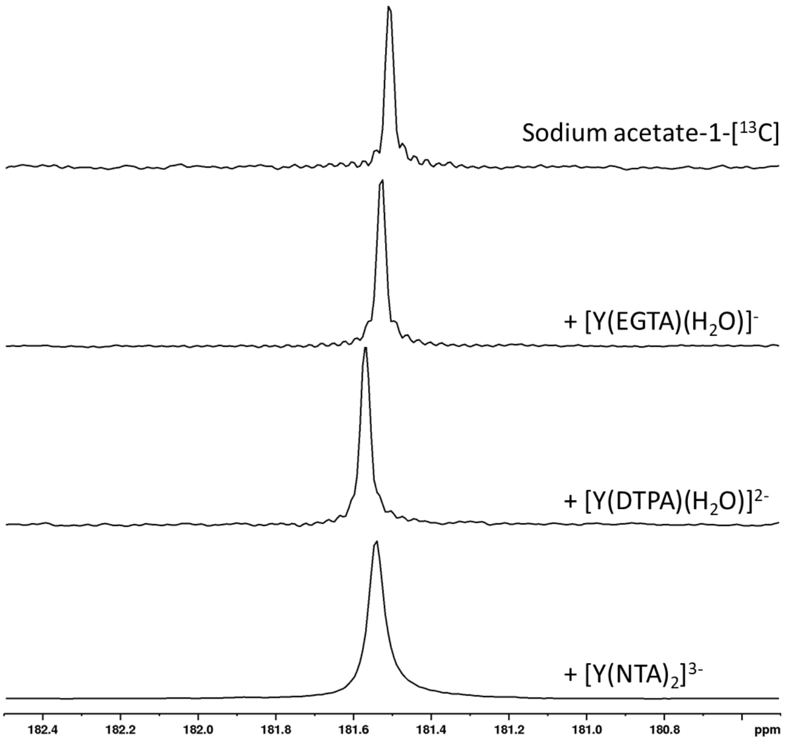

Figure S1: Partial ^{13}C NMR spectra of solutions of the indicated yttrium(III) polyaminocarboxylate complex (4 mM) with sodium acetate-1-[^{13}C] (1.5 equiv.) in D_2O (0.6 mL) recorded at 7 T and 298 K. The ^{13}C NMR spectrum of sodium acetate-1-[^{13}C] (1.5 equiv.) in D_2O (0.6 mL) is shown for reference. Spectra were not recorded with the same number of scans and are not shown on the same vertical scale. All spectra were processed using the same 1 Hz line broadening parameter.

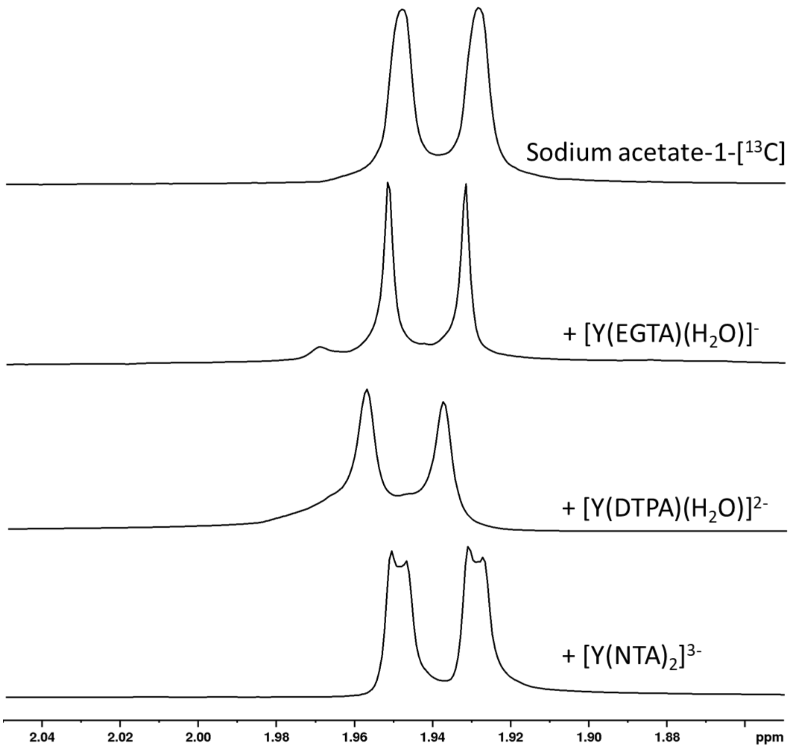

Figure S2: Partial ^{1}H NMR spectra of solutions of the indicated yttrium(III) polyaminocarboxylate complex (4 mM) with sodium acetate-1-[^{13}C] (1.5 equiv.) in D_2O (0.6 mL) recorded at 7 T and 298 K showing the signal for the acetate CH_3 group. The ^{1}H NMR spectrum of sodium acetate-1-[^{13}C] (1.5 equiv.) in D_2O (0.6 mL) is shown for reference. Spectra were not recorded with the same number of scans and are not shown on the same vertical scale. All spectra were processed using the same 0.3 Hz line broadening parameter.

Table S1: ^{13}C and ^{1}H chemical shift and peak width at half maximum of the acetate-[^{13}C] ^{13}C NMR peak when added (1.5 equiv.) to the indicated yttrium(III) polyaminocarboxylate complex (4 mM) in D_2O (0.6 mL). Note that width at half maximum has been rounded to the nearest 0.5 Hz.

**Complex**	**Acetate ^{13}C chemical shift / ppm**	**Acetate ^{13}C NMR peak width at half height / Hz**	**Acetate ^{1}H chemical shift / ppm**	**Acetate ^{1}H NMR peak width at half height / Hz**
Acetate (free)	181.51	2.0	1.94	2.0
$[Y(EGTA)(H_2O)]^{-}$	181.53	2.0	1.94	1.0
$[Y(DTPA)(H_2O)]^{2-}$	181.57	2.0	1.95	2.0
$[Y(NTA)_2]^{3}$	181.54	3.5	1.94	2.0

S1.2. Reaction with bicarbonate

The complexes $[Y(EGTA)(H_2O)]^{-}$, $[Y(DTPA)(H_2O)]^{2-}$ or $[Y(NTA)_2]^{3-}$ (4 mM) and sodium bicarbonate-[^{13}C] (1.5 equiv.) were dissolved in D_2O (0.6 mL) and left at 40 °C for 18 hours. ^{13}C NMR spectroscopy was used to examine the appearance of additional ^{13}C NMR signals, or signal broadening, to provide evidence for the *in situ* formation of ternary adducts. These spectra are shown in Figure S3 and are summarised in Table S2.

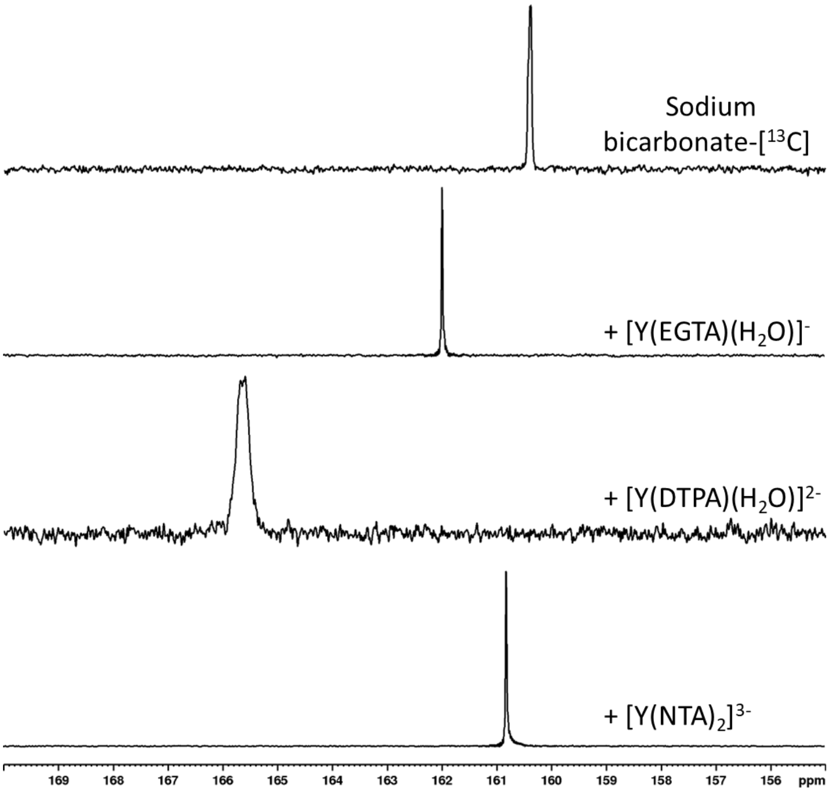

Figure S3: Partial ^{13}C NMR spectra of solutions of the indicated yttrium(III) polyaminocarboxylate complex (4 mM) with sodium bicarbonate-[^{13}C] (1.5 equiv.) in D_2O (0.6 mL) recorded at 7 T and 298 K. The ^{13}C NMR spectrum of sodium bicarbonate-[^{13}C] (1.5 equiv.) in D_2O (0.6 mL) is shown for reference. Spectra were not recorded with the same number of scans and are not shown on the same vertical scale. All spectra were processed using the same 1 Hz line broadening parameter.

Table S2: ^{13}C chemical shift and peak width at half maximum of the bicarbonate-[^{13}C] ^{13}C NMR peak when added (1.5 equiv.) to the indicated yttrium(III) polyaminocarboxylate complex (4 mM) in D_2O (0.6 mL). Note that width at half maximum has been rounded to the nearest integer number.

Complex	Bicarbonate ^{13}C chemical shift / ppm	Bicarbonate ^{13}C NMR peak width at half height / Hz
HCO_3^-	160.40	6
$[Y(EGTA)(H_2O)]^-$	161.99	2
$[Y(DTPA)(H_2O)]^{2-}$	165.68	20
$[Y(NTA)_2]^{3-}$	160.82	2

Samples of $[Y(EGTA)(H_2O)]^-$ and $[Y(DTPA)(H_2O)]^{2-}$ (4 mM) and sodium bicarbonate-[^{13}C] (1.5 equiv.) in D_2O (0.6 mL) were also examined using ^{13}C NMR spectroscopy at variable temperatures. These spectra are shown in Figure S4 and S5 respectively with the corresponding line widths of ^{13}C NMR signals presented in Table S3.

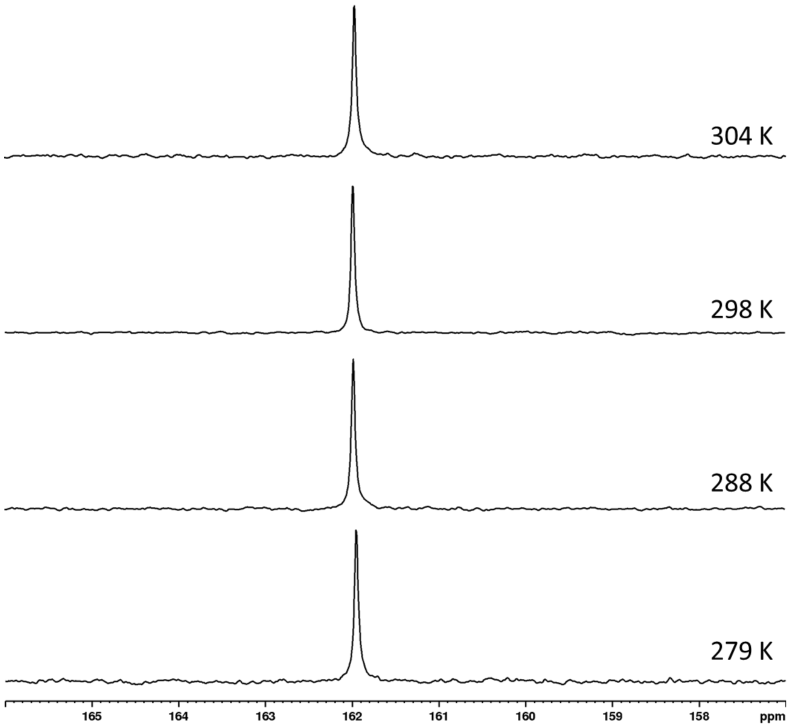

Figure S4: Partial ^{13}C NMR spectra of solutions of $[Y(EGTA)(H_2O)]^-$ (4 mM) with sodium bicarbonate-[^{13}C] (1.5 equiv.) in D_2O (0.6 mL) recorded at 7 T and the indicated temperature. Spectra were not recorded with the same number of scans and are not shown on the same vertical scale. All spectra were processed using the same 3 Hz line broadening parameter.

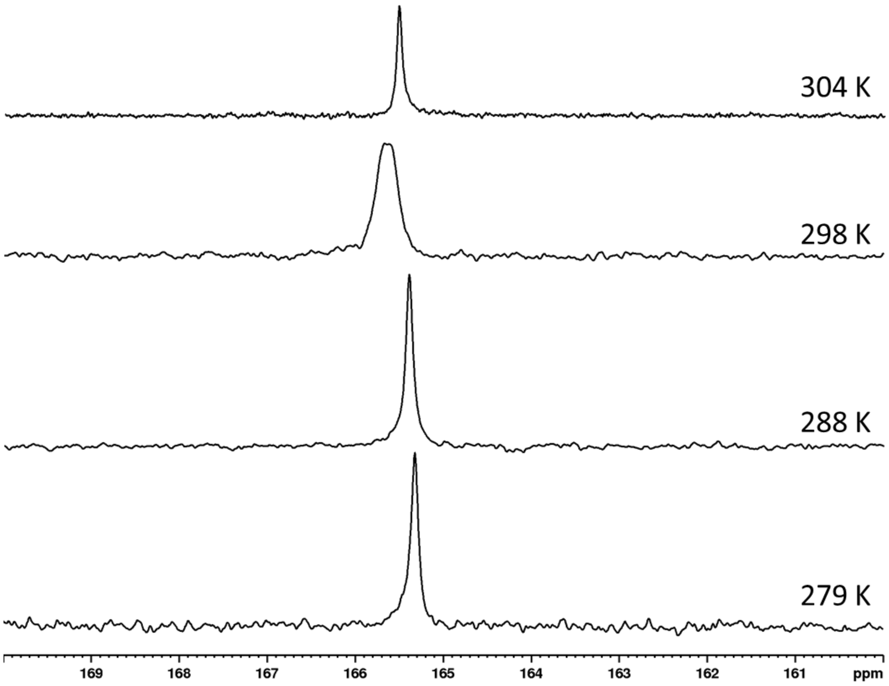

Figure S5 Partial ^{13}C NMR spectra of solutions of $[Y(DTPA)(H_2O)]^{2-}$ (4 mM) with sodium bicarbonate-[^{13}C] (1.5 equiv.) in D_2O (0.6 mL) recorded at 7 T and the indicated temperature. Spectra were not recorded with the same number of scans and are not shown on the same vertical scale. All spectra were processed using the same 3 Hz line broadening parameter.

Table S3: ^{13}C peak width at half maximum of the bicarbonate-[^{13}C] ^{13}C NMR peak as a function of temperature for samples of the indicated yttrium(III) polyaminocarboxylate complex (4 mM) and sodium bicarbonate-[^{13}C] (1.5 equiv.) in D_2O (0.6 mL). Note that width at half maximum has been rounded to the nearest integer number.

**Complex**	**Bicarbonate ^{13}C NMR peak width at half height / Hz**			
	**279 K**	**288 K**	**298 K**	**304 K**
$[Y(EGTA)(H_2O)]^{-}$	4	4	2	4
$[Y(DTPA)(H_2O)]^{2-}$	8	7	20	5

S1.3. Reaction with pyruvate

The complexes [Y(EGTA)(H_2O)]$^{-}$, [Y(DTPA)(H_2O)]$^{2-}$ and [Y(NTA)$_2$]$^{3-}$, (4 mM) and sodium pyruvate-1-[^{13}C] (1.5 equiv.) were dissolved in methanol-*d*$_4$ (0.6 mL) and left at 40 °C for 18 hours. ^{13}C and ^{1}H NMR spectroscopy was used to examine the appearance of additional ^{13}C or ^{1}H NMR signals, or signal broadening, to provide evidence for the *in situ* formation of ternary adducts. These spectra are shown in Figure S6 and S7 respectively.

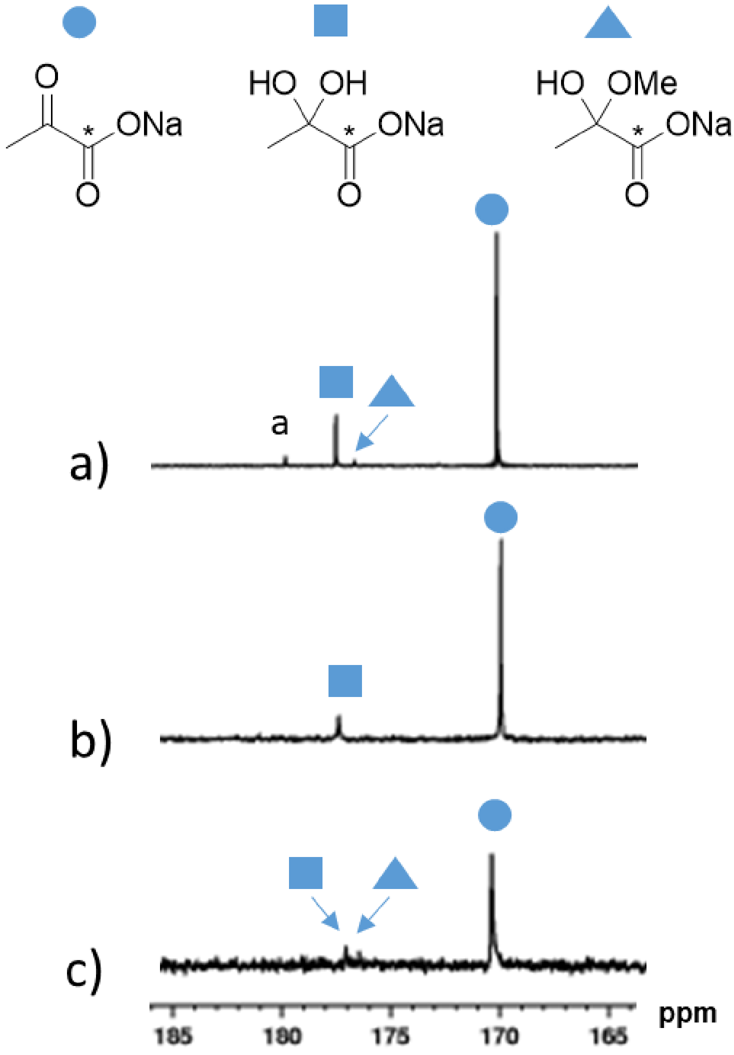

Figure S6: Partial ^{13}C NMR spectra of solutions of a) [Y(EGTA)(H_2O)]$^{-}$ b) [Y(DTPA)(H_2O)]$^{2-}$ and c) [Y(NTA)$_2$]$^{3-}$ (4 mM) with sodium pyruvate-1-[^{13}C] (1.5 equiv.) in methanol-*d*$_4$ (0.6 mL) recorded at 7 T at 298 K. Spectra were not recorded with the same number of scans and are not shown on the same vertical scale. All spectra were processed using the same 1 Hz line broadening parameter. Signals labelled a are consistent with isomers of parapyruvic acid.

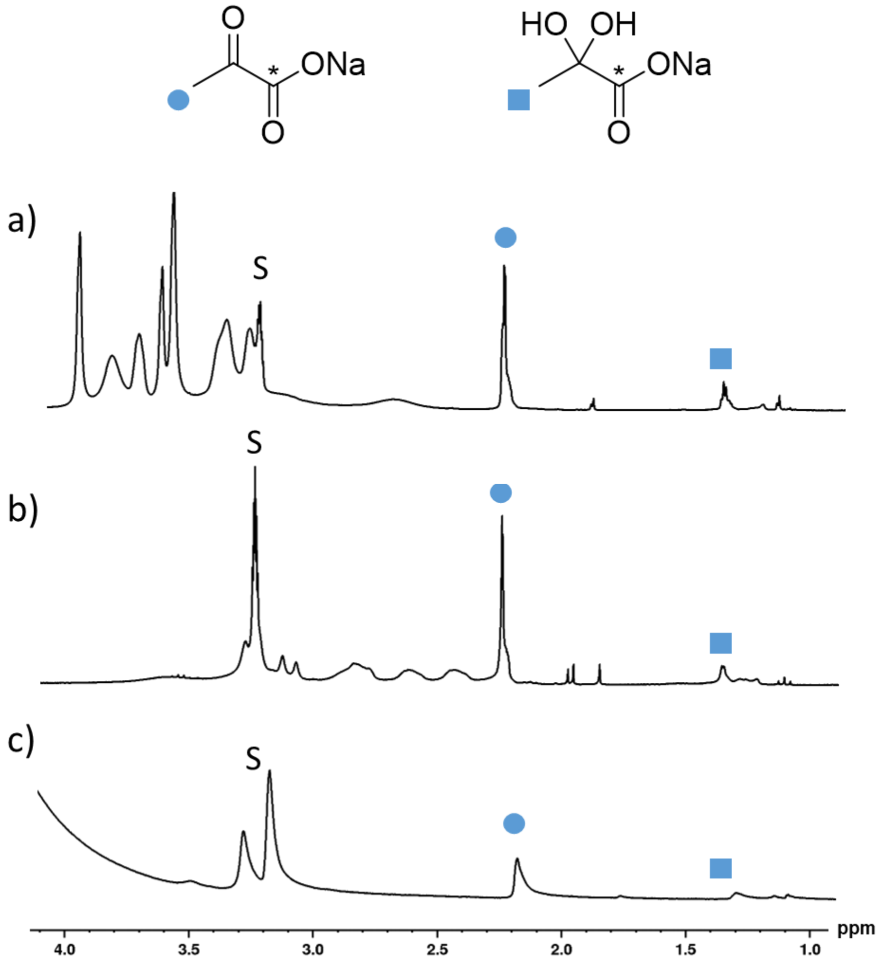

Figure S7: Partial ^{1}H NMR spectra of solutions of a) $[Y(EGTA)(H_2O)]^{-}$ [b) $[Y(DTPA)(H_2O)]^{2-}$ c) $[Y(NTA)_2]^{3-}$ and (4 mM) with sodium pyruvate-1-[^{13}C] (1.5 equiv.) in methanol-*d*4 (0.6 mL) recorded at 7 T at 298 K. Spectra were not recorded with the same number of scans and are not shown on the same vertical scale. All spectra were processed using the same 0.3 Hz line broadening parameter. Unassigned signals likely correspond to sites within the polyaminocarboxylate ligand.

S2. [Y(DO3A)(H_2O)$_2$]: *in situ* ternary adduct formation with acetate, bicarbonate and pyruvate

S2.1. Reaction with acetate

[Y(DO3A)(H_2O)$_2$] (4 mM) and sodium acetate-1-[^{13}C] (1.5 equiv.) were dissolved in D_2O (0.6 mL) and left at 40 ^{o}C for 18 hours. ^{13}C and ^{1}H NMR spectroscopy was used to examine the appearance of additional NMR signals, or signal broadening, to provide evidence for the *in situ* formation of ternary adducts. ^{13}C NMR spectra are shown in the main paper and are summarised in Table S4. ^{1}H NMR spectra are shown in Figure S8.

Table S4: ^{13}C and ^{1}H chemical shift and peak width at half maximum of the acetate-[^{13}C] ^{13}C NMR peak when added (1.5 equiv.) to [Y(DO3A)(H_2O)$_2$] (4 mM) in D_2O (0.6 mL). Note that width at half maximum has been rounded to the nearest 0.5 Hz.

Complex	Acetate ^{13}C chemical shift / ppm	Acetate ^{13}C NMR peak width at half height / Hz	Acetate ^{1}H chemical shift / ppm	Acetate ^{1}H NMR peak width at half height / Hz
Free Acetate	181.51	2.0	1.94	2.0
[Y(DO3A)(H_2O)$_2$]	181.97	5.5	1.96	2.5

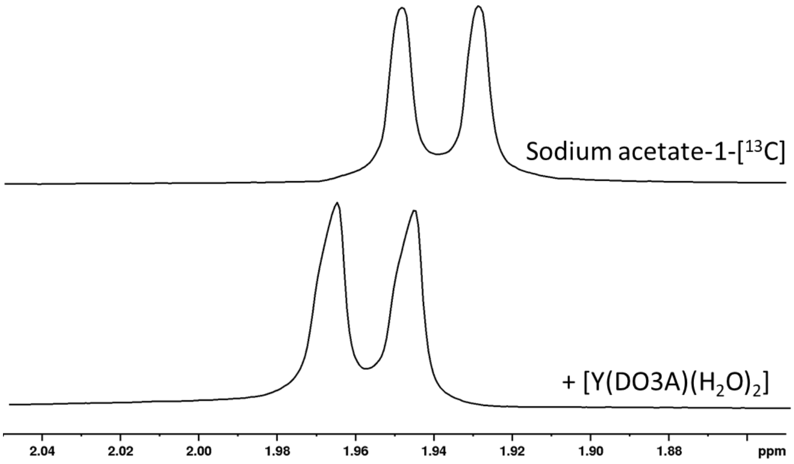

Figure S8: Partial ^{1}H NMR spectra of solutions of [Y(DO3A)(H_2O)$_2$] (4 mM) with sodium acetate-1-[^{13}C] (1.5 equiv.) in D_2O (0.6 mL) recorded at 7 T and 298 K with reference spectra of sodium acetate-1-[^{13}C] (1.5 equiv.) in D_2O (0.6 mL). Reference spectra were not recorded with the same number of scans and are not shown on the same vertical scale.

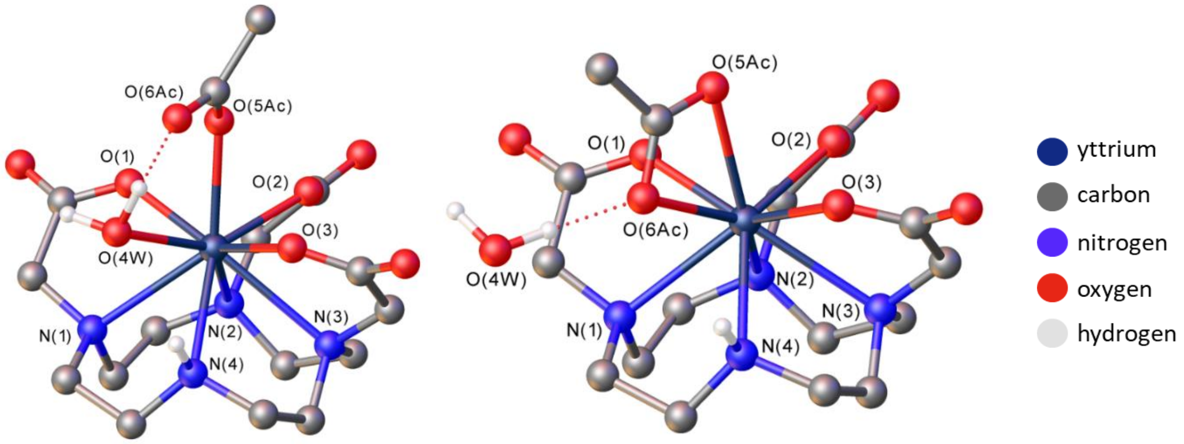

Figure S9: Structures of the $[Y(DO3A)(CH_3COO)]^{-}\cdot H_2O$ system obtained with DFT calculations showing either monodentate (left) or bidentate (right) acetate binding. Bidentate binding with displacement of the water molecule to the second coordination sphere leads to a more stable structure, with a relative Gibbs free energy of -12.3 kJ mol^{-1}. The bond distances (Å) of the Y(III) coordination sphere in the structure with bidentate binding are as follows: Y-N(1), 2.630; Y-N(2), 2.620; Y-N(3), 2.646; Y-N(4), 2.586; Y-O(1), 2.327; Y-O(2), 2.286; Y-O(3), 2.314; Y-O(5Ac), 2.485;Y -O(6Ac), 2.414. Many ^{1}H atoms have been omitted for visual clarity.

S2.2. Reaction with bicarbonate

$[Y(DO3A)(H_2O)_2]$ (4 mM) and sodium bicarbonate-[^{13}C] (1.5 equiv.) were dissolved in D_2O (0.6 mL) and left at 40 ^{o}C for 18 hours. ^{13}C NMR spectroscopy (example in main paper, summarised in Table S5) was used to examine the appearance of additional ^{13}C NMR signals, or signal broadening, to provide evidence for the *in situ* formation of ternary adducts. ^{13}C NMR spectroscopy was also recorded at variable temperatures (Figure S10, summarised in Table S6).

Table S5: ^{13}C chemical shift and peak width at half maximum of the bicarbonate-[^{13}C] ^{13}C NMR peak when added (1.5 equiv.) to $[Y(DO3A)(H_2O)_2]$ (4 mM) in D_2O (0.6 mL). Note that width at half maximum has been rounded to the nearest integer number.

Complex	Bicarbonate ^{13}C chemical shift / ppm	Bicarbonate ^{13}C NMR peak width at half height / Hz
HCO_3^-	160.40	6
$[Y(DO3A)(H_2O)_2]$	160.96	55

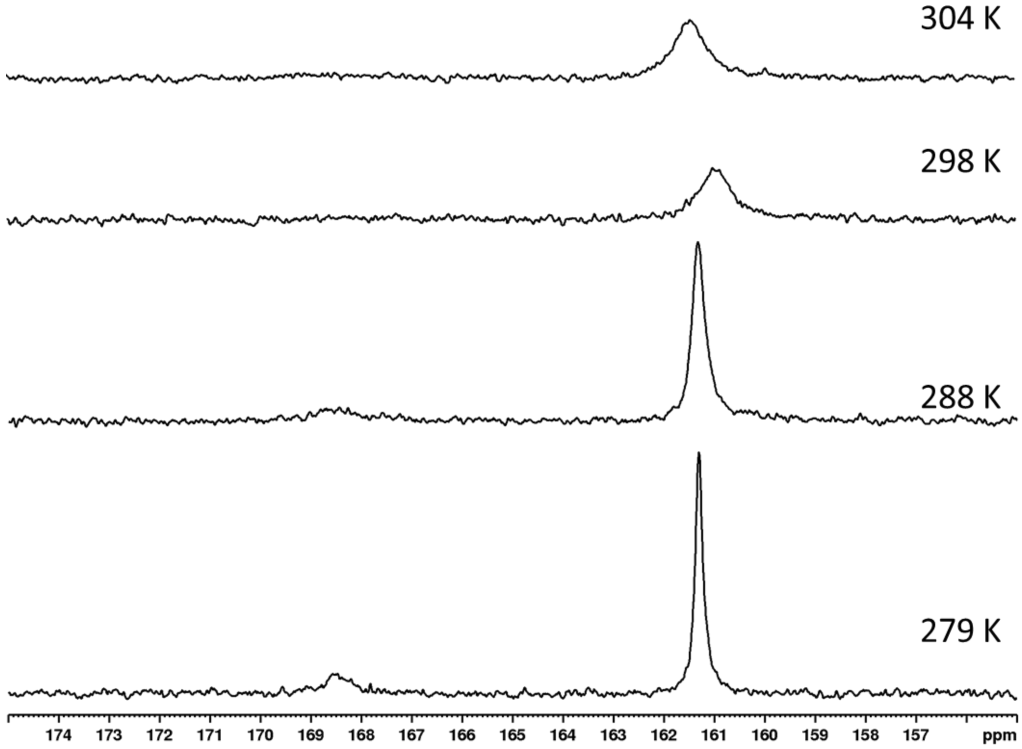

Figure S10: Partial ^{13}C NMR spectra of solutions of [Y(DO3A)(H_2O)$_2$] (4 mM) with sodium bicarbonate-[^{13}C] (1.5 equiv.) in D_2O (0.6 mL) recorded at 7 T and the indicated temperature. Spectra were not recorded with the same number of scans and are not shown on the same vertical scale. All spectra were processed using the same 3 Hz line broadening parameter.

Table S6: ^{13}C peak width at half maximum of the bicarbonate-[^{13}C] ^{13}C NMR peak as a function of temperature for samples of the indicated yttrium(III) polyaminocarboxylate complex (4 mM) and sodium bicarbonate-[^{13}C] (1.5 equiv.) in D_2O (0.6 mL). Note that width at half maximum has been rounded to the nearest integer number.

Complex	Bicarbonate ^{13}C NMR peak width at half height / Hz			
	279 K	288 K	298 K	304 K
[Y(DO3A)(H_2O)$_2$]	13<br>40*	24<br>90*	55	54

*an additional peak at *ca* δ 168.5 appears at low temperature with the line width denoted by the asterisk

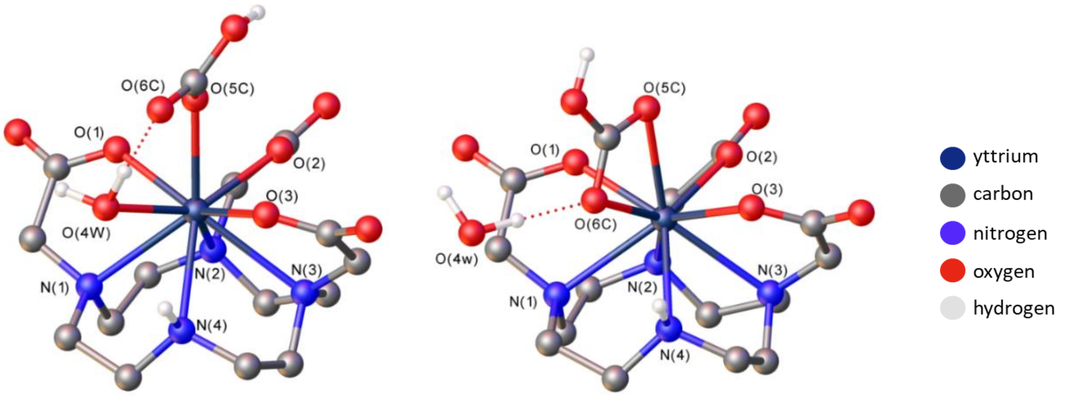

Figure S11: Structures of the $[Y(DO3A)(HCO_3)]^{-}\cdot H_2O$ system obtained with DFT calculations showing either monodentate (left) or bidentate (right) bicarbonate binding. Bidentate binding with displacenment of the water molecule to the second coordination sphere leads to a more stable structure, with a relative Gibbs free energy of -5.0 kJ mol^{-1}. The bond distances (Å) of the Y(III) coordination sphere in the structure with bidentate binding are as follows: Y-N(1), 2.623; Y-N(2), 2.614; Y-N(3), 2.636; Y-N(4), 2.586; Y-O(1), 2.321; Y-O(2), 2.282; Y-O(3), 2.311; Y-O(5C), 2.515;Y-O(6C), 2.419. Many ^{1}H atoms have been omitted for visual clarity.

S2.3. Reaction with pyruvate

$[Y(DO3A)(H_2O)_2]$ (4 mM) and sodium pyruvate-1-[^{13}C] (1.5 equiv.) were dissolved in D_2O (0.6 mL) and left at 40 ^{o}C for 18 hours. ^{13}C NMR spectroscopy was used to examine the *in situ* formation of ternary adducts (see main paper). DFT calculations were used to optimise a geometry for a $[Y(DO3A)(pyruvate)]^{-}$ adduct indicated in ^{13}C NMR experiments (structure given in main paper). This structure is also shown in Figure S12 with the indicated bond distances calculated by DFT studies.

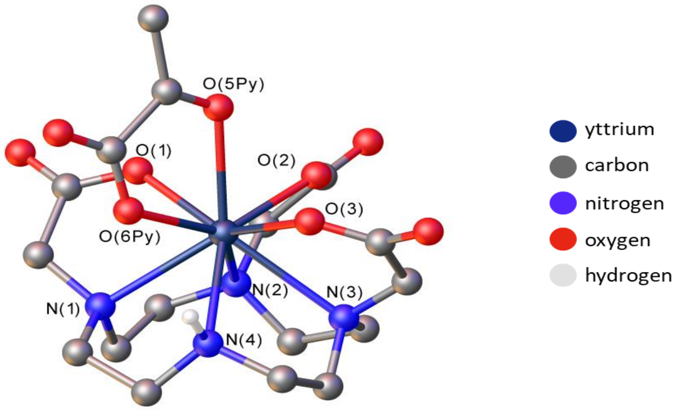

Figure S12: Structure of the $[Y(DO3A)(pyruvate)]^{-}$ system obtained with DFT calculations. Bond distances (Å) of the Y(III) coordination sphere: Y-N(1), 2.624; Y-N(2), 2.631; Y-N(3), 2.644; Y-N(4), 2.576; Y-O(1), 2.322; Y-O(2), 2.299; Y-O(3), 2.307; Y-O(5Py), 2.521;Y-O(6Py), 2.360. Many ^{1}H atoms have been omitted for visual clarity.

S3. Behaviour of $[Y(EDTA)(H_2O)_q]^-$ with bicarbonate and pyruvate: Activation of pyruvate by $[Y(EDTA)(H_2O)_q]^-$

S3.1. Reaction with acetate

$[Y(EDTA)(H_2O_2)_q]^-$ (4 mM) and sodium acetate-1-[^{13}C] (1.5 equiv.) were dissolved in D_2O (0.6 mL) and left at 40 °C for 18 hours. ^{13}C, and to a lesser extent ^{1}H, NMR spectroscopy was used to examine the appearance of additional NMR signals, or signal broadening, to provide evidence for the *in situ* formation of ternary adducts. These spectra are shown in Figure S13 and are summarised in Table S7.

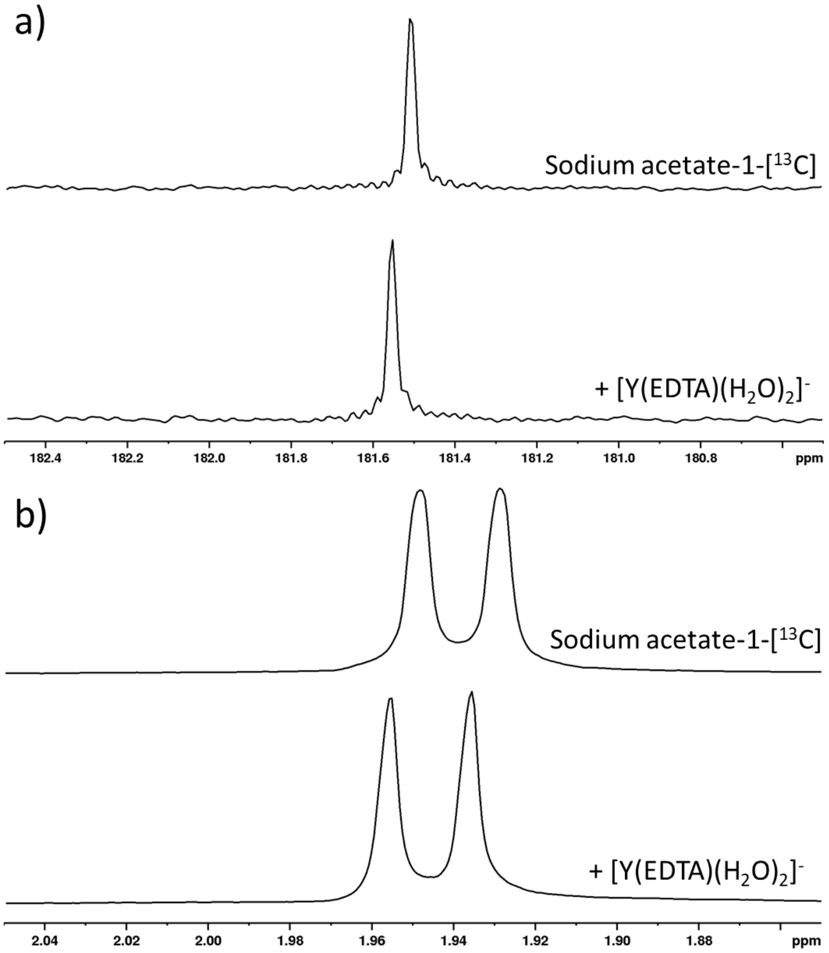

Figure S13: Partial a) ^{13}C and b) ^{1}H NMR spectra of solutions of $[Y(EDTA)(H_2O)_q]^-$ (4 mM) with sodium acetate-1-[^{13}C] (1.5 equiv.) in D_2O (0.6 mL) recorded at 7 T and 298 K with reference spectra of sodium acetate-1-[^{13}C] (1.5 equiv.) in D_2O (0.6 mL). Reference spectra were not recorded with the same number of scans and are not shown on the same vertical scale.

Table S7: ^{13}C and ^{1}H chemical shift and peak width at half maximum of the acetate-[^{13}C] ^{13}C NMR peak when added (1.5 equiv.) to the indicated yttrium(III) polyaminocarboxylate complex (4 mM) in D_2O (0.6 mL). Note that width at half maximum has been rounded to the nearest 0.5 Hz.

Complex	Acetate ^{13}C chemical shift / ppm	Acetate ^{13}C NMR peak width at half height / Hz	Acetate ^{1}H chemical shift / ppm	Acetate ^{1}H NMR peak width at half height / Hz
Free Acetate	181.51	2.0	1.94	2.0
$[Y(EDTA)(H_2O)_q]^-$	181.55	2.0	1.95	1.5

S3.2. Reaction with bicarbonate

$[Y(EDTA)(H_2O)_q]^-$ (4 mM) and sodium bicarbonate-[^{13}C] (1.5 equiv.) were dissolved in D_2O (0.6 mL) and left at 40 ^oC for 18 hours. ^{13}C NMR spectroscopy (example in main paper, summarised in Table S8) was used to examine the appearance of additional ^{13}C NMR signals, or signal broadening, to provide evidence for the *in situ* formation of ternary adducts. ^{13}C NMR spectroscopy was also recorded at variable temperatures (Figure S14, summarised in Table S9).

Table S8: ^{13}C chemical shift and peak width at half maximum of the bicarbonate-[^{13}C] ^{13}C NMR peak when added (1.5 equiv.) to $[Y(EDTA)(H_2O)_q]^-$ (4 mM) in D_2O (0.6 mL). Note that width at half maximum has been rounded to the nearest integer number.

Complex	Bicarbonate ^{13}C chemical shift / ppm	Bicarbonate ^{13}C NMR peak width at half height / Hz
HCO_3^-	160.40	6
$[Y(EDTA)(H_2O)_q]^-$	163.81	150

Figure S14: Partial ^{13}C NMR spectra of solutions of $[Y(EDTA)(H_2O)_q]^-$ (4 mM) with sodium bicarbonate-[^{13}C] (1.5 equiv.) in D_2O (0.6 mL) recorded at 7 T and the indicated temperature. Spectra were recorded with the same number of scans and are shown on the same vertical scale. All spectra were processed using the same 3 Hz line broadening parameter.

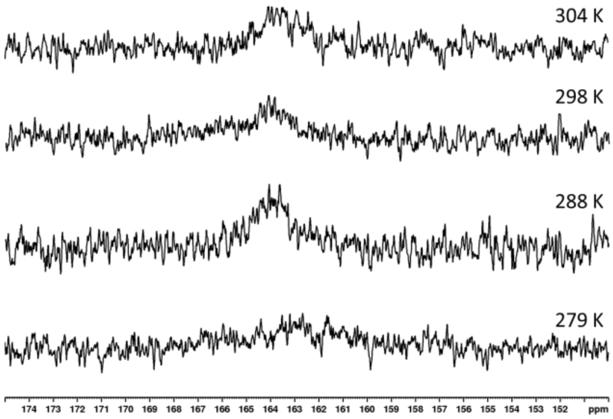

Table S9: ^{13}C peak width at half maximum of the bicarbonate-[^{13}C] ^{13}C NMR peak as a function of temperature for samples of $[Y(EDTA)(H_2O)_q]^-$ (4 mM) and sodium bicarbonate-[^{13}C] (1.5 equiv.) in D_2O (0.6 mL). Note that width at half maximum has been rounded to the nearest integer number.

**Complex**	**Bicarbonate ^{13}C NMR peak width at half height /Hz**			
	**279 K**	**288 K**	**298 K**	**304 K**
$[Y(EDTA)(H_2O)_q]^-$	420	200	150	180

DFT calculations were used to optimise a geometry for a $[Y(EDTA)(HCO_3)]^{2-}$ adduct indicated in ^{13}C NMR experiments (structure given in main paper). This structure is also shown in Figure S15 with the indicated bond distances calculated by DFT studies.

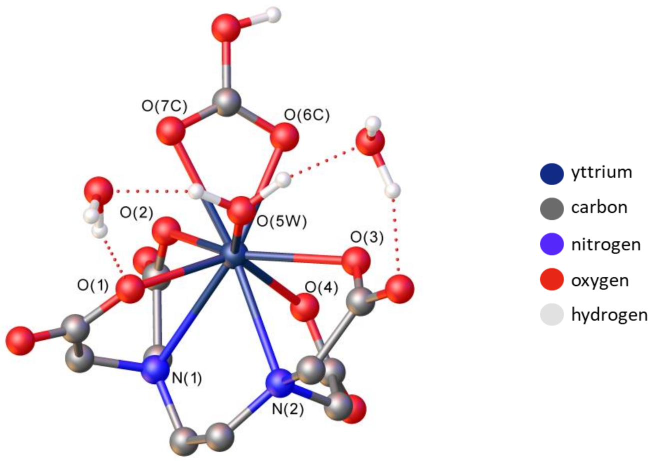

Figure S15: Structure of the $[Y(EDTA)(HCO_3)]^{2-}\cdot 3H_2O$ system obtained with DFT calculations. Bond distances (Å) of the Y(III) coordination sphere: Y-N(1), 2.752; Y-N(2), 2.587; Y-O(1), 2.328; Y-O(2), 2.335; Y-O(3), 2.314; Y-O(4), 2.436; Y-O(5W), 2.453; Y-O(6C), 2.392;Y -O(7C), 2.531. Many ^{1}H atoms have been omitted for visual clarity.

S3.3. Reaction with pyruvate

Samples containing [Y(EDTA)(H_2O)$_q$]$^-$ (4 mM) and sodium pyruvate-1-[^{13}C] (1.5 equiv.) were dissolved in methanol-d_4 (0.6 mL) and left at 40 °C for 18 hours. ^{1}H NMR spectroscopy was used to examine the activation of pyruvate to form acetic acid, CO_2 and HCO_3^- products, these are shown in Figure S16. Corresponding ^{13}C NMR measurements are presented in the main paper.

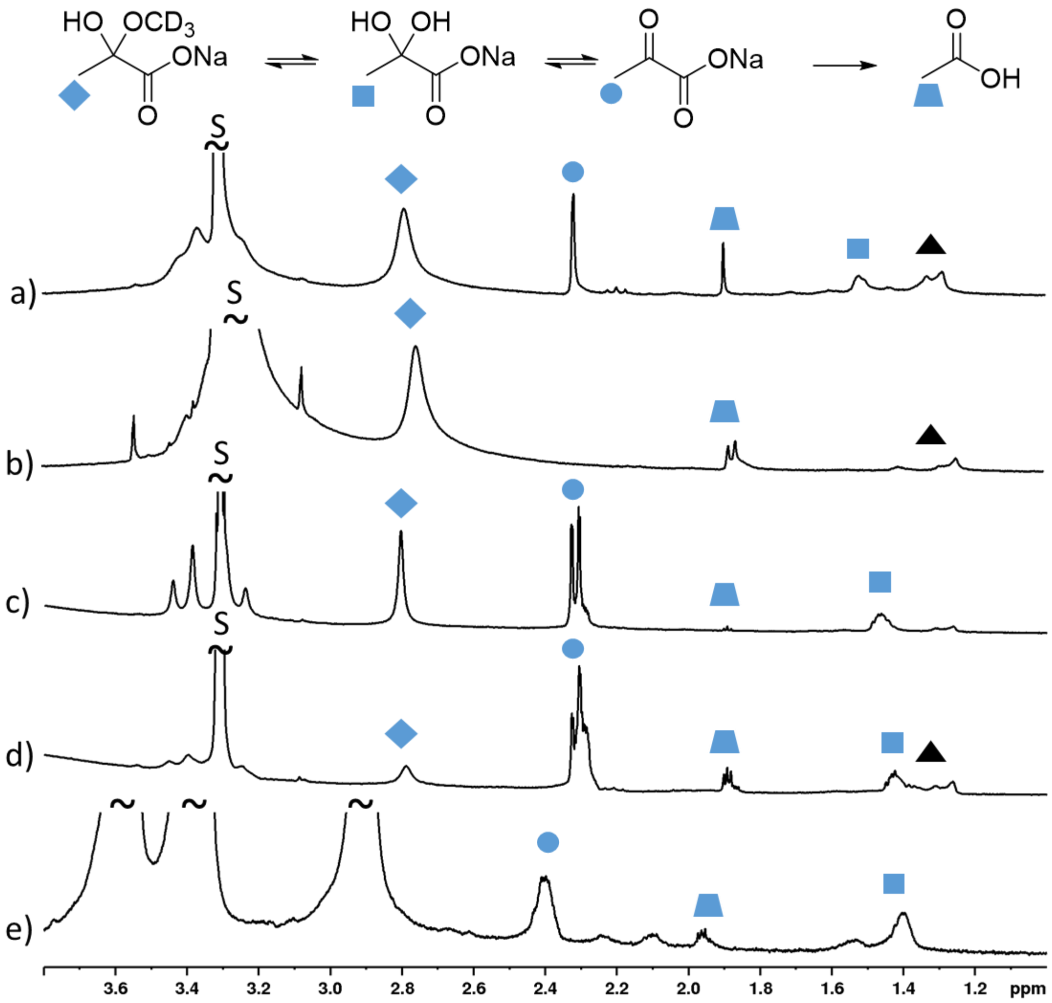

Figure S16: Partial ^{1}H NMR spectra of [Y(EDTA)(H_2O)$_q$]$^-$ (4 mM) and a) sodium pyruvate-1-[^{13}C] or b) sodium pyruvate-1,2-[$^{13}C_2$] (1.5 equiv.) in methanol-d_4 after 18 hours at 338 K c)-d) sodium pyruvate-1,2-[$^{13}C_2$] (1.5 equiv.) in methanol-d_4 after c) ca 1 hour and d) *ca*. 3 hours at 298 K and e) sodium pyruvate-1,2-[$^{13}C_2$] (1.5 equiv.) in D_2O after 3 hrs at 298 K. All spectra were recorded at 298 K and 7 T and are processed with the same 0.3 Hz line broadening parameter. Spectra were not recorded with the same number of scans and are not shown on the same vertical scale. Signals assigned with the black triangles correspond to an isomer of parapyruvic acid. Signals denoted with squares have been assigned as pyruvate hydrate, although it is possible that these correspond to pyruvate hemiacetal.

DFT calculations were used to optimise a geometry for a $[Y(EDTA)(pyruvate)(H_2O)]^{2-}$ adduct indicated in ^{13}C NMR experiments (structure given in main paper). This structure is also shown in Figure S17 with the indicated bond distances calculated by DFT studies. The structure of an analogous $[Y(EDTA)(pyruvate\text{-}hydrate)]^{2-}$ adduct is shown in Figure S18.

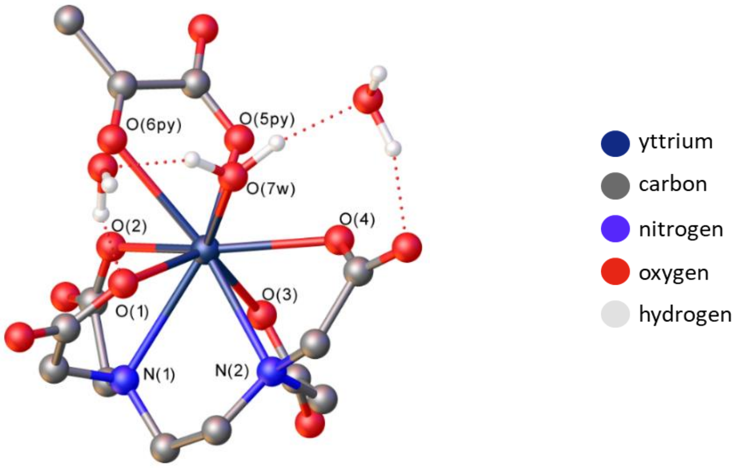

Figure S17: Structure of the $[Y(EDTA)(pyruvate)(H_2O)]^{2-}\cdot 2H_2O$ system obtained with DFT calculations. Bond distances (Å) of the Y(III) coordination sphere: Y-N(1), 2.743; Y-N(2), 2.594; Y-O(1), 2.336; Y-O(2), 2.343; Y-O(3), 2.315; Y-O(4), 2.428 Y-O(5py), 2.330; Y-O(6Py), 2.568;Y -O(7w), 2.439. Many ^{1}H atoms have been omitted for visual clarity.

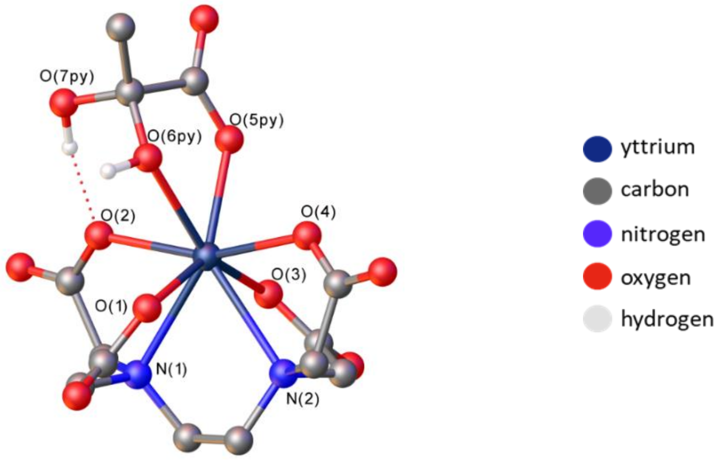

Figure S18: Structure of the $[Y(EDTA)(pyruvate\text{-}hydrate)]^{2-}$ system obtained with DFT calculations. Bond distances (Å) of the Y(III) coordination sphere: Y-N(1), 2.601; Y-N(2), 2.594; Y-O(1), 2.287; Y-O(2), 2.343; Y-O(3), 2.277; Y-O(4), 2.297 Y-O(5py), 2.312; Y-O(6Py), 2.445. Many ^{1}H atoms have been omitted for visual clarity.

S3.4. Control experiments: Reaction of yttrium salts with pyruvate

Samples containing $Y(NO_3)_3 \cdot xH_2O$ or $YCl_3 \cdot xH_2O$, (4 mM) and sodium pyruvate-1-[^{13}C] (1.5 equiv.) were dissolved in methanol-d_4 (0.6 mL) and left at 40 °C for 18 hours. ^{13}C NMR spectroscopy was used to examine if any activation of pyruvate occurs (Figure S19). These spectra reveal that CO_2 and HCO_3^- products are not formed, although various ^{13}C NMR signals are observed. These do not correspond to free pyruvate and it is expected that they correspond to inorganic yttrium pyruvate salts.

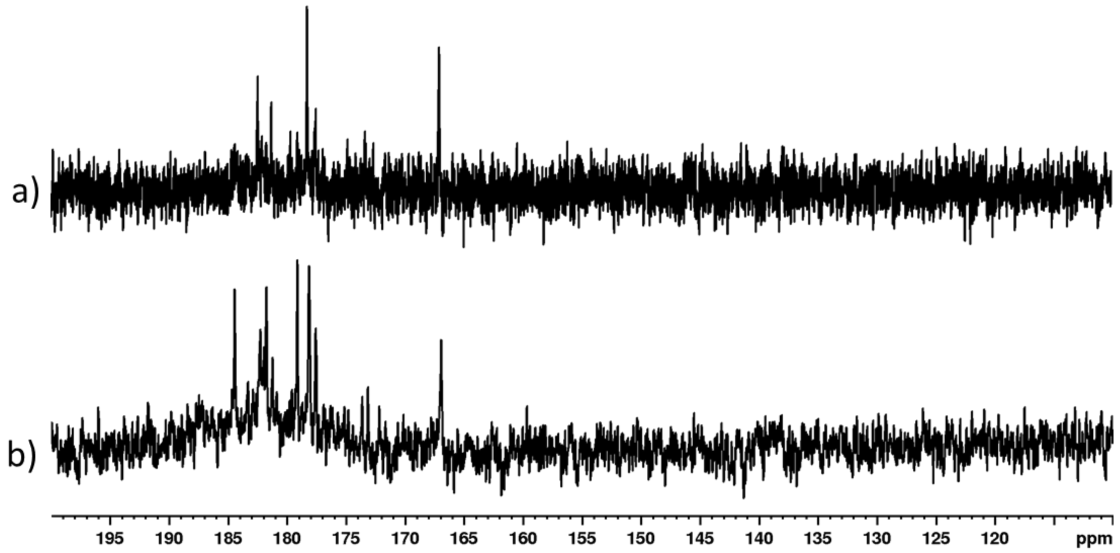

Figure S19: Partial ^{13}C NMR spectra of sodium pyruvate-1-[^{13}C] (1.5 equiv.) and a) $Y(NO_3)_3 \cdot xH_2O$ (4 mM) or b) $YCl_3 \cdot xH_2O$ (4 mM) in methanol-d_4 after 18 hours at 338 K recorded at 298 K and 7 T. Spectra are not shown on the same vertical scale and were processed with the same 3 Hz line broadening parameter.

S4. Probing pyruvate activation by $[Y(EDTA)(H_2O)_q]^-$ using SABRE-hyperpolarised ^{13}C NMR

This reaction between $[Y(EDTA)(H_2O)_q]^-$ and sodium pyruvate was investigated using SABRE-hyperpolarised pyruvate. Solutions of [IrCl(COD)(IMes)] (where COD = *cis*,*cis*-1,5-cyclooctadiene) and IMes = 1,3-bis(2,4,6-trimethyl-phenyl)imidazol-2-ylidene) (final concentration 5 mM), dimethyl sulfoxide (final concentration 30 mM) and sodium pyruvate-1-[$^{13}C_2$] (final concentration 30 mM) in methanol-d_4 (0.5 mL) were reacted with H_2 (3 bar) for 30 mins at room temperature to form $[Ir(H)_2(\kappa^2$-pyruvate)(DMSO)(IMes)] *in situ*.[1–3] The NMR tube containing this solution was then shaken with pH_2 (3 bar) for 30 seconds in a mu metal shield to produce pyruvate in an enhanced nuclear spin state. After this 30 second shaking period, the lid of the NMR tube was removed and $[Y(EDTA)(H_2O)_q]^-$ (final concentration 3 mM) in methanol-d_4 (0.1 mL) was added. This addition was performed with the NMR tube still in the mu metal shield and the tube was shaken for *ca* 1 second inside the shield to enable adequate mixing of the two components. The sample was then placed quickly into the 9.4 T spectrometer for analysis by single scan ^{13}C NMR. In these spectra, antiphase signals are produced as a consequence of the formation of singlet order which is more efficient at these polarisation transfer fields than single spin order transfer to the enriched site. This topic has been covered in previous works[2] and can result in more intense ^{13}C NMR signals for the 1.1% naturally abundant sodium pyruvate-1,2-[$^{13}C_2$] isotopologue (Figure S20a).

When larger concentrations (25 mM) of $[Y(EDTA)(H_2O)_q]^-$ were added hyperpolarsed ^{13}C pyruvate NMR signals were quenched (Figure S20b). This is attributed to competitive binding of pyruvate to both iridium and yttrium centres which favours the latter at high yttrium concentrations. This limits the reversible exchange between pyruvate and iridium that is necessary to observe the SABRE effect.

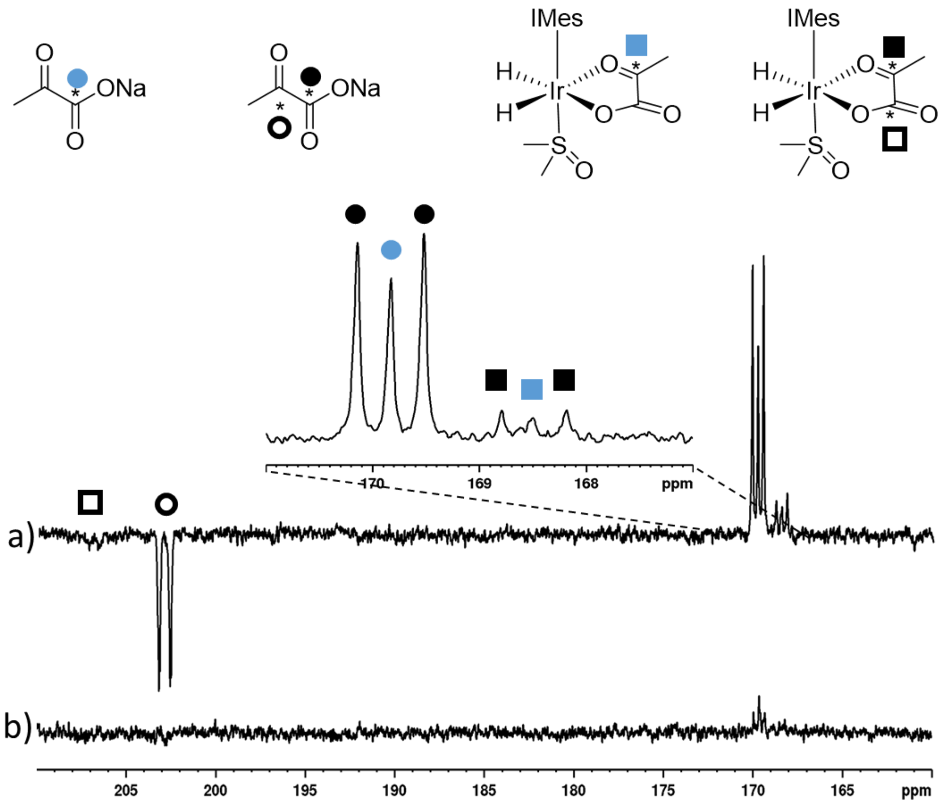

Figure S20: Partial single scan ^{13}C NMR spectra of a) SABRE hyperpolarised sodium pyruvate-1-[^{13}C] (30 mM) with [IrCl(COD)(IMes)] (5 mM) and dimethyl sulfoxide (30 mM) in methanol-d_4 (0.6 mL) after shaking with pH_2 (3 bar) for 30 seconds in a mu metal shield and b) analogous spectrum when $[Y(EDTA)(H_2O)_q]^-$ (25 mM) in methanol-d_4 (0.1 mL) is added to the solution from a) following fresh shaking with pH_2. Spectra are recorded at 9.4 T and 298 K. Signals marked by the asterisk denote a ^{13}C labelled site, those in the pyruvate 2 position are naturally abundant.

S4.1. Control Experiments: SABRE hyperpolarisation of disodium bicarbonate-[^{13}C]

Control experiments were performed to determine whether Na_2CO_3 can become hyperpolarised *via* SABRE. A solution of [IrCl(COD)(IMes)] (where COD = *cis*,*cis*-1,5-cyclooctadiene and IMes = 1,3-bis(2,4,6-trimethyl-phenyl)imidazol-2-ylidene) (5 mM), methylphenyl sulfoxide (50 mM) and disodium carbonate-[^{13}C] (30 mM) in methanol-d_4 (0.6 mL) were reacted with H_2 (3 bar) for 30 mins at room temperature. Thermally polarised ^1H NMR measurements revealed the formation of hydride-containing complexes (Figure S21a). Upon shaking the sample with 3 bar pH$_2$ for 10 seconds in a mu metal shield, signals for a hyperpolarised metal complex were observed with ALTADENA appearance at δ −23.43 and −29.79 (Figure S21b). These resonance are indicative of chemical environments *trans* to chloride and oxygen respectively[2,4,5] and is likely of the form [IrCl(H)$_2$(IMes)(*O*-L$_1$)(L$_2$)] where L is dimethylsulfoxide, H_2O or CD_3OD. Unfortunately, this species could not be characterised by 2D NMR spectroscopy at 245 K: no NOE connections from these hydride resonances to other ^1H sites were discerned. When the shaking process repeated and single scan ^{13}C NMR spectra recorded, no enhanced ^{13}C NMR signals are observed. This suggests that under the conditions employed in the sample containing sodium pyruvate and [Y(EDTA)(H$_2$O)$_q$]$^-$, hyperpolarisation of the HCO_3^- resonance is a result of rapid reaction from hyperpolarised pyruvate, rather than reversible interaction of the HCO_3^- product with the iridium SABRE catalyst.

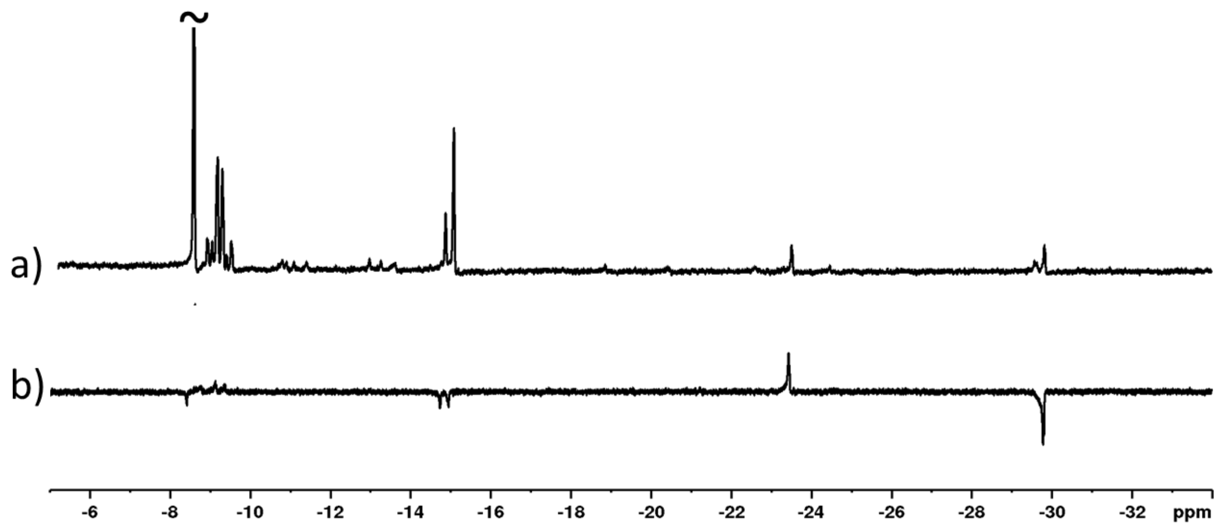

Figure S21: a) Partial 32 scan ^1H NMR spectra of the hydride region when samples containing [IrCl(COD)(IMes)] (5 mM), methylphenylsulfoxide (10 eq.) and Na_2CO_3 (4 eq.) in methanol-d_4 (0.6 mL) are activated with 3 bar H_2 for 1 hour at room temperature. b) Partial single scan hyperpolarised ^1H NMR spectra of the hydride region when the sample from a) was shaken with 3 bar pH$_2$ for 10 seconds at 6.5 mT.

S5. References

(1) Tickner, B. J.; Lewis, J. S.; John, R. O.; Whitwood, A. C.; Duckett, S. B. Mechanistic Insight into Novel Sulfoxide Containing SABRE Polarisation Transfer Catalysts. *Dalton Trans.* **2019**, *48* (40), 15198–15206.

(2) Iali, W.; Roy, S. S.; Tickner, B. J.; Ahwal, F.; Kennerley, A. J.; Duckett, S. B. Hyperpolarising Pyruvate through Signal Amplification by Reversible Exchange (SABRE). *Angew. Chem.* **2019**, *131* (30), 10377–10381.

(3) Tickner, B. J.; Semenova, O.; Iali, W.; Rayner, P. J.; Whitwood, A. C.; Duckett, S. B. Optimisation of Pyruvate Hyperpolarisation Using SABRE by Tuning the Active Magnetisation Transfer Catalyst. *Cat. Sci. Technol.* **2020**, *10*, 1343–1355.

(4) Fekete, M.; Roy, S. S.; Duckett, S. B. A Role for Low Concentration Reaction Intermediates in the Signal Amplification by Reversible Exchange Process Revealed by Theory and Experiment. *Phys. Chem. Chem. Phys.* **2020**, *22* (9), 5033–5037.

(5) Knecht, S.; Hadjiali, S.; Barskiy, D. A.; Pines, A.; Sauer, G.; Kiryutin, A. S.; Ivanov, K. L.; Yurkovskaya, A. V.; Buntkowsky, G. Indirect Detection of Short-Lived Hydride Intermediates of Iridium N-Heterocyclic Carbene Complexes via Chemical Exchange Saturation Transfer Spectroscopy. *J. Phys. Chem. C.* **2019**, *123* (26), 16288–16293.
